# High-throughput phenotyping with deep learning gives insight into the genetic architecture of flowering time in wheat

**DOI:** 10.1093/gigascience/giz120

**Published:** 2019-11-19

**Authors:** Xu Wang, Hong Xuan, Byron Evers, Sandesh Shrestha, Robert Pless, Jesse Poland

**Affiliations:** 1 Department of Plant Pathology, Kansas State University, 4024 Throckmorton PSC, 1712 Claflin Road, Manhattan, KS 66506, USA; 2 Department of Computer Science, George Washington University, 4000 Science and Engineering Hall, 800 22nd Street NW, Washington, DC 20052, USA

**Keywords:** convolutional neural network, deep learning, genetic architecture, plant breeding, wheat

## Abstract

**Background:**

Measurement of plant traits with precision and speed on large populations has emerged as a critical bottleneck in connecting genotype to phenotype in genetics and breeding. This bottleneck limits advancements in understanding plant genomes and the development of improved, high-yielding crop varieties.

**Results:**

Here we demonstrate the application of deep learning on proximal imaging from a mobile field vehicle to directly estimate plant morphology and developmental stages in wheat under field conditions. We developed and trained a convolutional neural network with image datasets labeled from expert visual scores and used this “breeder-trained” network to classify wheat morphology and developmental stages. For both morphological (awned) and phenological (flowering time) traits, we demonstrate high heritability and very high accuracy against the “ground-truth” values from visual scoring. Using the traits predicted by the network, we tested genotype-to-phenotype association using the deep learning phenotypes and uncovered novel epistatic interactions for flowering time. Enabled by the time-series high-throughput phenotyping, we describe a new phenotype as the rate of flowering and show heritable genetic control for this trait.

**Conclusions:**

We demonstrated a field-based high-throughput phenotyping approach using deep learning that can directly measure morphological and developmental phenotypes in genetic populations from field-based imaging. The deep learning approach presented here gives a conceptual advancement in high-throughput plant phenotyping because it can potentially estimate any trait in any plant species for which the combination of breeder scores and high-resolution images can be obtained, capturing the expert knowledge from breeders, geneticists, pathologists, and physiologists to train the networks.

## Background

Limitations in phenotyping are widely recognized as a critical constraint in genetic studies and in plant breeding [1, 2]. Initial developments in field-based, high-throughput phenotyping (HTP) have focused on direct sensor or image measurements to extract proxies for traits of interest such as vegetation indexes from spectral reflectance [3, 4] or plant height from digital elevation models [5]. While lending great insight to plant processes, this first generation of HTP is limited in assessment of “complex” traits such as plant morphology or growth stage that cannot be assessed by a linear function of pixel values. While these complex morphological and developmental features are readily distinguished by a trained eye, the assessment of these phenotypes with high-throughput platforms is challenging, particularly under field conditions used in plant breeding programs.

Deep learning has emerged as a powerful machine learning approach that takes advantage of both the extraordinary computing power and very large datasets that are often now available [6]. Deep learning bypasses the need to explicitly define which features are most useful or needed for data analysis. Instead deep learning optimizes a complete end-to-end process to map data samples to outputs that are consistent with the large, labeled datasets used for training the network. For image analysis tasks, convolutional neural networks (CNNs) learn this end-to-end mapping by optimizing for many layers of filters. The first filters are easily interpreted as low-level image features (e.g., detecting edges, bright points, or color variations), and subsequent layers are increasingly complicated combinations of earlier features. When there is sufficient training data, CNNs dramatically outperform all alternative existing methods for image analysis. For benchmark classification tasks attempting to label which of 1,000 different objects are in an image, results have increased from 84.6% in 2012 [7]to 96.4% in 2015 [8].

On the basis of this impressive performance of the latest CNNs, these deep learning approaches are being applied to challenging tasks in plant phenomics [8] including root and shoot feature identification [9], leaf counting [10, 11], classifying types of biotic and abiotic stress [12], counting seeds per pod [13], and detection of wheat spikes [14]. Initial studies have shown a wide array of potential applications for CNNs in plant phenomics. With increasingly robust image datasets, the use of CNNs has great potential for accurate estimation of plant phenotypes directly from images.

One challenge that has emerged in using CNNs for plant phenomics is the development of suitable datasets that are sufficiently annotated for training the networks [8, 9]. The labeling of images, particularly when going from weak to stronger annotations, is a time- and resource-intensive constraint for the future of plant phenomics [10]. While it is a greater challenge to train networks with weaker annotations, the size of these datasets can be greatly expanded beyond what is tractable with extensive manual annotation. Furthermore, approaches that can generate a large number of images with imputed labels from visual scoring as demonstrated in this study have potential to exponentially increase the size and scope of labeled image datasets for the phenomics community.

Given the broad applications and demonstrated success of deep learning, we hypothesized that this deep learning approach could be a powerful tool for estimating phenotypes of interest directly from images in segregating plant populations under field conditions used by breeding programs. Such implementations of HTP would have direct application to improve the efficiency of plant breeding while being directly relevant to the phenotypes of interest to breeders and the sizes of populations used by these breeding programs. When applied in a relevant context at the scale of entire breeding programs (e.g., thousands to tens of thousands of field plots), these phenomics tools can contribute to accelerated development of high-yielding, climate-resilient new varieties.

## Data Description

### Field-based high-throughput imaging of wheat plots

**Figure 1:**
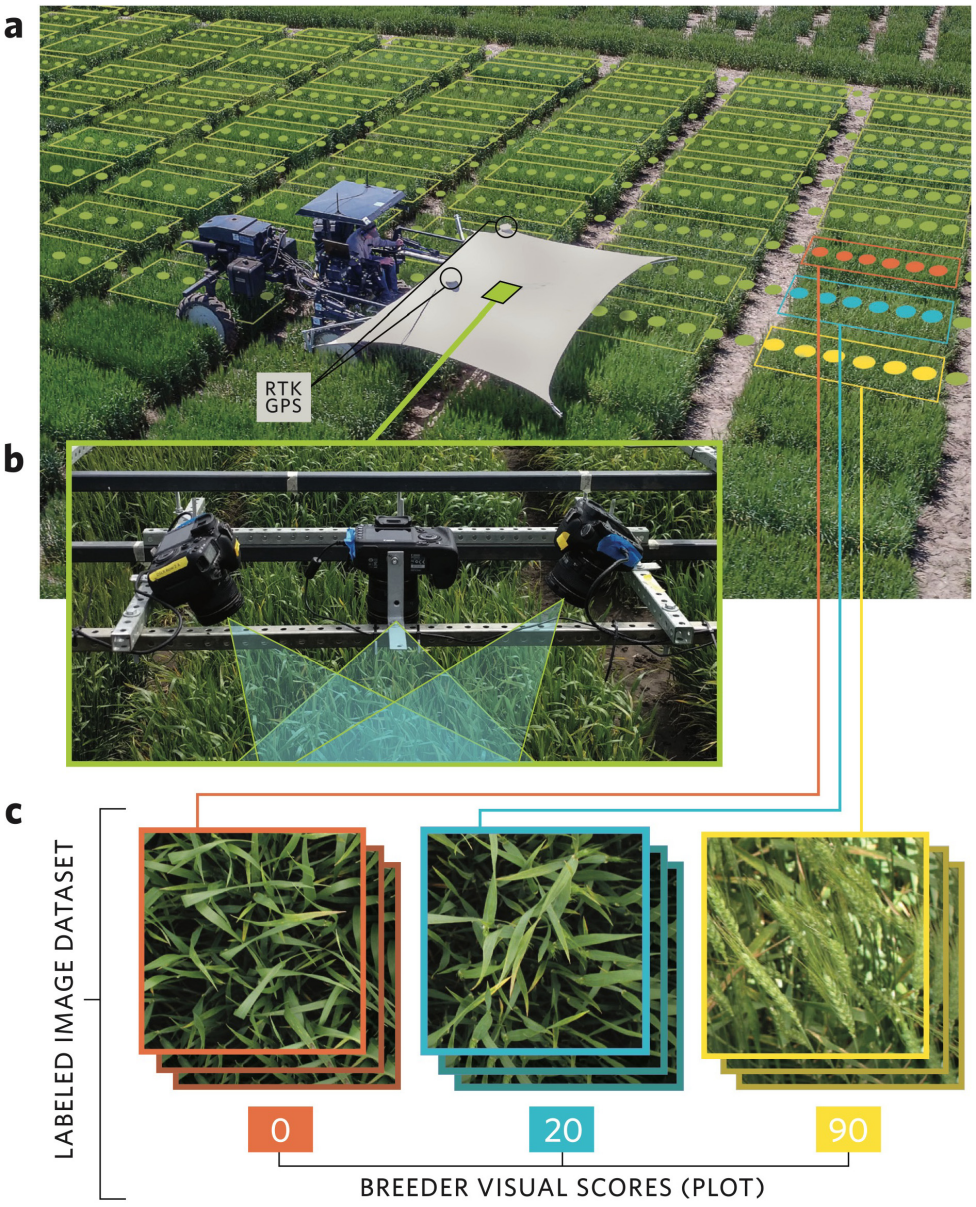
Phenotyping platform and “breeder-trained” image datasets in this study. (**a**) Aerial view of field-based high-throughput phenotyping platform deployed in present study traversing wheat plots with superimposed example representation of imaging positions and example field plot boundaries. (**b**) imaging array of multiple DSLR cameras deployed on the phenotyping platform to collect geo-referenced proximal images of the wheat canopy. (**c**) combination of images assigned to respective field plots and merged with visual breeder scored to develop the labeled imaged dataset for training the neural networks.

To advance high-throughput phenotyping of complex morphological and developmental traits under field conditions, we developed a high-clearance field vehicle [15] equipped with an array of DSLR cameras collecting geo-positioned images (Fig. 1a and b). This platform was deployed across wheat field trials in 2016 and 2017. Each year we grew 2 trials, (i) a recombinant inbred line (RIL) population from a cross between wheat cultivars “Lakin” and “Fuller” and (ii) a panel of diverse historical and modern winter wheat varieties consisting of a total of 1,398 plots each year. We captured >400,000 proximal images of the wheat canopies throughout the growing seasons in 2016 and 2017. These images were geo-referenced and 135,771 and 139,752 of the images were assigned to individual field plots in 2016 and 2017, respectively, on the basis of surveyed coordinates of the field plots and geo-tagged images (Fig. 1, Supplementary Fig. S1). This approach enabled high-throughput proximal imaging on an individual plot level (1.5 m × 2.4 m plot size). Concordant with imaging, field plots were visually scored for percent heading and spike morphology of awned or awnless. To generate a large collection of labeled images suitable for deep learning while avoiding time-consuming manual annotations, the images from a given plot were labeled with the “breeder scores” of awned/awnless and percentage heading visually assessed at the same time points on the same respective plots as the image data collection (Fig. 1c). The labeled image dataset(s) collected and analyzed in this study are available in the GigaDB repository [16].

## Analysis

### Development of convolutional neural networks

To assess plant features that cannot be measured directly by sensors with the high-throughput platform, we developed a CNN network that could be trained using these geo-positioned images that are labeled with visual scores and subsequently automatically classify and estimate the phenotypes of interest. As a starting point, we first approached the qualitative trait of awn morphology (Supplementary Fig. S2).

**Figure 2:**
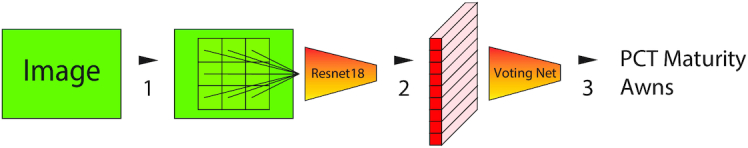
Schematic of the “WheatNet” neural network developed for classifying cropped image patches followed by census voting network for the whole image.

An initial challenge in the development of the CNN was memory constraints that limit the networks to analyzing relatively small images, but the images were captured at very high resolution. Because the relevant image features are quite small (e.g., wheat awns at 1–2 mm width), reducing the size of the image would make these features invisible. We therefore cropped the images into a 3 × 3 grid of 9 patches of 224 × 224 pixel size. To build the full “WheatNet,” we then extended the CNN architecture that analyzes images with a small additional network that combined features from the 9 patches to create a consensus estimate for the image (Fig. 2).

We used this developed CNN architecture in a training-validation-testing approach to predict the awn phenotype in the diverse panel of inbred lines in which there were awned and awnless variants. The training and validation images were from this diversity panel evaluated in 2017 with 700 plots, of which 29 plots were awnless and 671 were awned. Model training used 2,000 images for awned plots and 1,800 images for awnless plots. As a validation dataset, we sampled 70 plots from the awned and 5 plots from the awnless and left the remaining plots as the training data. We validated the WheatNet on a set of 300 images each from awned and awnless plots. On this set, the network classification matched the visual score at 99.2% on the training set and 98.6% on the validation set.

To test the WheatNet for predicting awn morphology, we applied the network trained with data from 2017 to test images from field trials of the diversity panel in 2016, which contained 12,504 images from 675 awned plots and 32 awnless plots. At the level of individual images, the prediction accuracy was 98.9% for awned and 98.7% for awnless phenotypes (Supplemental Table S1). Because many images were captured for each plot, we applied a plot-level consensus voting, which increased the accuracy to 99.7% for awned and 100% for awnless. Strikingly, we observed that only 2 plots were inconsistent between visual scoring and the CNN predictions and that these 2 plots were the same variety (“MFA-2018”) across both field replications. Further inspection showed that this variety was a heterogeneous “atypical awnlette” phenotype (Supplementary Fig. S2), indicating that the CNN was able to detect subtle atypical phenotypes that were lost or ignored in the human scoring.

### Measurement of percentage heading

Observing initial proof of concept for using deep learning to score a simple morphological trait with 2 classes, we extended this approach to a more complex problem of developmental phenotypes using time-series imaging. Flowering time is a critical trait under intense selection in natural populations and breeding programs. Owing to tightly closed flowers in wheat, spike emergence (heading time or heading date) is used as a close proxy for flowering time in breeding and genetics studies. To estimate the heading date of wheat, which is classically defined as the date in which spikes (ears) have emerged from 50% of the tillers [17], we applied the CNN to classify percentage heading over the longitudinal image datasets. For the initial trait estimation of percent heading we used the same CNN architecture and training approach as was used for predicting the wheat awn phenotype and trained the network with 2 years of image data from the diversity panel. An additional feature of predicting percentage heading makes this problem different from standard classification problems, in that the breeder labels are given in 10% increments, and there is some inconsistency in these labels. To address this, we modified the algorithm that trains the CNN to give partial credit for predictions that are within 10% and 20% from the assigned label.

On the classification problem for percent heading the network prediction exactly matched the 10 percentile classifications from visual assessment on 45.12% (training set) and 41.27% (validation set) of the images, which is much better than a random guess at 9.10% (1 out of 11 classes). The confusion matrix for training, validation, and testing the CNN for predicting percent heading showed clear diagonal patterns, indicating linear consistency between the observed and predicted values (Supplemental Fig. S3). Although there was lower accuracy in the testing phase, the diagonal linear pattern remained consistent. From these observations, we had strong evidence that the CNN is accurately estimating percentage heading for images across the range of heading values and throughout the season. Following this conclusion, we applied the CNN trained on the diversity panel to predict percentage heading and calculate heading date for a biparental RIL population and determine whether genotype-to-phenotype associations could be detected directly from phenotypes estimated through deep learning.

**Figure 3:**
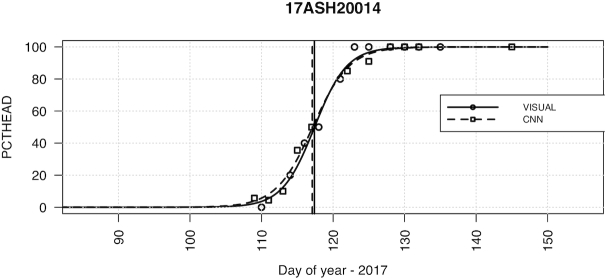
Example of logistic regression to score heading date from time-series measurements for a single field plot (17ASH20014). Shown are visual scores (circles) and predictions from the convolutional neural network (squares) and the respective fitted regression lines. The 50% interaction of the regression was identified and scored as the heading date.

**Figure 4:**
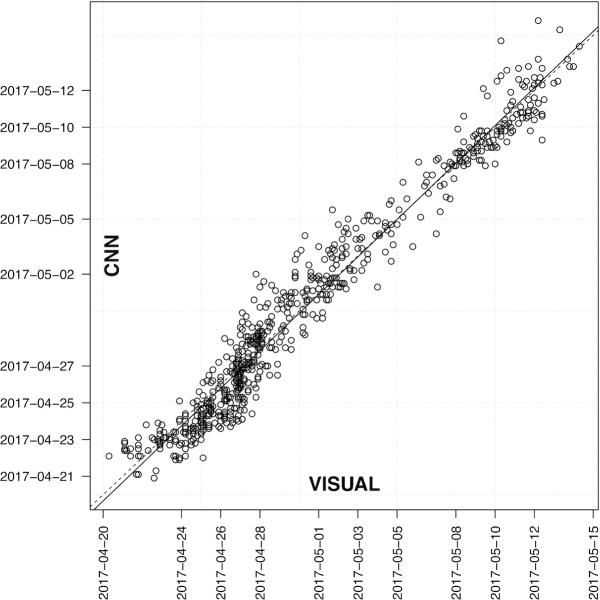
Heading date compared between visual scoring and convolutional neural network (CNN) from logistic regression applied individually to 676 field plots in “Lakin × Fuller” recombinant inbred line population in 2017 for percentage heading from time-series observations (visual scoring) and time-series imaging (CNN predictions). The dates of visual scoring (VISUAL) and imaging data collection (CNN) are shown on the axes.

To translate the time-series imaging and CNN phenotypic predictions into a single time point for heading date, we applied a logistic regression to the percent heading measurements for each individual plot (Fig. 3). From the logistic regression fit, we then found the date intersecting the regression at 50% and assigned this time point as the heading date commensurate with the classical definition of 50% of heads emerged from the boot. Applying this logistic regression individually to each plot we obtained heading dates from the CNN predictions that were highly accurate compared with the heading date measured directly from visual scoring (Fig. 4). We observed that >57% and >88% of heading date measurements were within 1 and 2 days, respectively, with a mean absolute error of 0.99 and root mean square error of 1.25 days. The slope of the regression between the visual and CNN measurements of 1.02 indicated lack of bias from the CNN predictions. Reflecting accurate phenotypes under strong genetic control, the broad-sense heritability for heading date was very high when measured by both visual scoring (*H*^2^ = 0.982) and CNN predictions (*H*^2^ = 0.987).

An interesting novel phenotype that can be assessed with this time-series approach is the developmental rate of flowering (heading) progression within an inbred line. This measure of the rate of heading is derived from the slope of the logistic regression. Measuring the slope for each inbred line, we found heritable genetic variance for the rate of heading (*H*^2^_VISUAL_ = 0.621, *H*^2^_CNN_ = 0.514), indicating that this developmental rate phenotype is also under genetic control. Because the rate of heading might simply be an artifact from the heading date per se, we tested the correlation between timing and rate of heading and found weak negative correlation (*r* = −0.19, *P-*value < 0.001). Looking at RILs within the normal early range of heading date (e.g., before May 5; Day 125) we found no significant correlation (*r* = 0.078, *P-*value = 0.079), suggesting that the rate of heading is indeed under independent genetic control from heading date.

### Genetic analysis of flowering time

**Figure 5:**
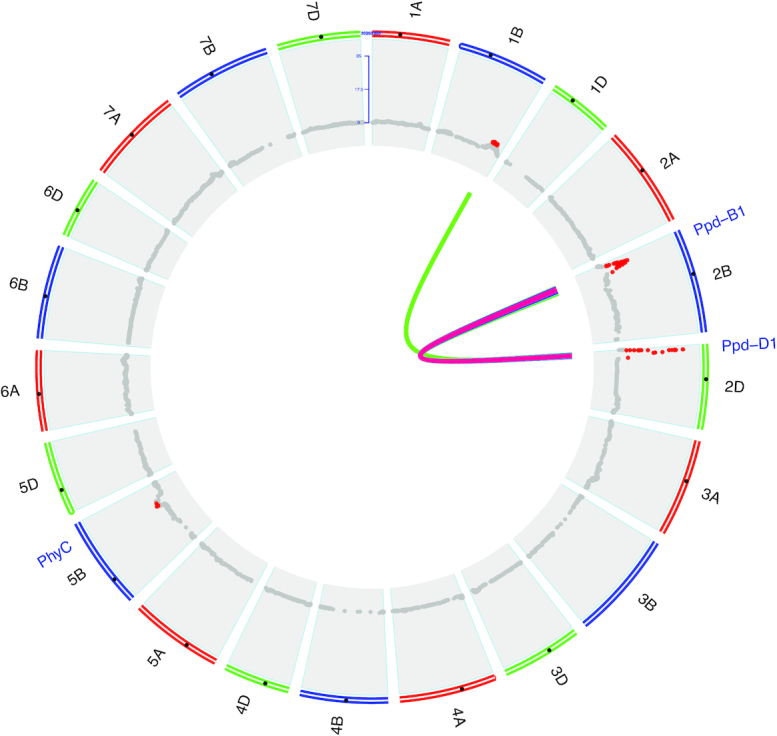
Genetic analysis of heading date scored by deep learning. Genome-wide association testing for heading date measured by deep learning on the Lakin × Fuller recombinant inbred line testing for 21 wheat chromosomes. Markers above Bonferroni multiple-test correction threshold shown in red. Epistatic interactions (internal connections) for 2-gene interaction model. Significant interactions at Bonferroni correction shown with heat colors indicating significance level (logarithm of the odds range from 4.97 [blue] to 22.4 [red]).

**Figure 6:**
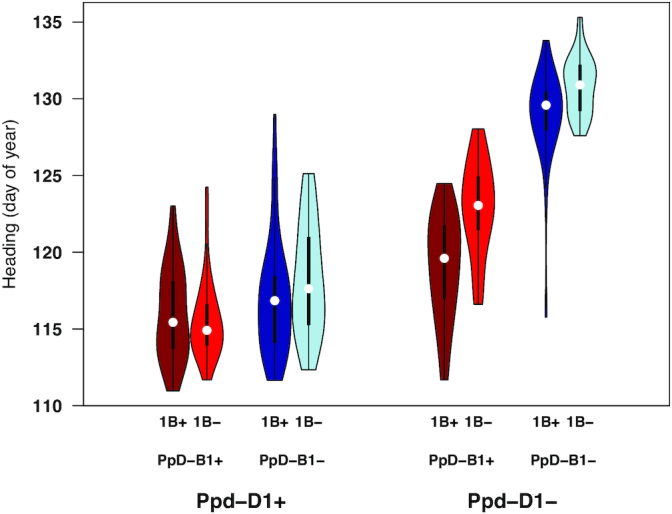
Epistatic interaction of loci controlling heading date. Phenotypic distributions of genotypes at loci showing significant epistatic interactions for heading date measured by CNN predictions including *Ppd-D1, Ppd-B1*, and QTL identified on chromosome 1B in the Lakin × Fuller RIL population. Plus and minus signs indicate genotypes with or without the early allele, respectively. In the violin plots, the white dots indicate the median, the thick bars indicate the interquartile range, and the thin bars indicate 1.5× the interquartile range.

Following the measurement of heading date using the neural network, we sought to determine the utility of phenotypes estimated directly from deep learning to uncover the genetic basis of the variation in flowering time present in the biparental population. Although the parental lines “Lakin” and “Fuller” are very similar in heading, the progeny showed vast transgressive segregation and also segregation distortion, indicating some underlying epistatic gene action (Supplementary Fig. S4). We implemented a genome-wide scan of 8,237 genotyping-by-sequencing markers and found strong associations for *PpD-D1* and *PpD-B1* as well as associations on 5B and a novel quantitative trait locus (QTL) positioned at the distal end of chromosome 1B (Fig. 5). Suspecting epistatic gene action based on the phenotypic distribution, we tested all significant markers for putative epistatic interactions and found strong interactions between *PpD-D1* and *Ppd-B1* as well as between *PpD-D1* and the locus on 1B (Fig. 5). Interestingly, the modeling of the interactions between all 3 loci removed the main effect of the 1B locus per se, with this locus having an opposite effect in the presence of *PpD-D1* early (insensitive) allele (Fig. 6).

While we found heritable genetic variance for the rate of heading, we were not able to find any genetic association within this population (Supplementary Fig. S5). Implicating missing heritability for the rate of flowering suggested a diffuse genetic architecture of many small effect alleles. We therefore tested whole-genome polygenic models (BayesA and G-BLUP) to capture the genetic variance for the rate of heading. We ran 100 replications of cross-validation predicting 10% masked phenotypes and were able to model 18–25% of the heritable genetic variance.

## Discussion

From this study, we have demonstrated that deep learning with “breeder-trained” neural networks from proximal field-based imaging can accurately classify plant morphology. When applied to time-series image datasets this approach can likewise accurately predict developmental stages such as flowering time. Furthermore, these machine vision phenotypes can be used directly to uncover genetic determinants in the populations, connecting genotype to phenotype in the same way as classical approaches to phenotyping.

An important advancement of the approach presented here is that there is no additional time investment in developing the labeled image set for training. Many applications applying deep learning for image-based phenotyping require extensive annotation of the training image sets, such as annotating plant features of interest prior to network training [9, 18]. As demonstrated in this study, HTP image datasets from field trials can be labeled through direct imputation of visual scores routinely collected in the field by breeders. This approach can be used to develop very large labeled image datasets for training networks on any phenotype of interest without any further human input.

Although the images are labeled, and hence the network subsequently trained, by experienced individuals, there are inherent limitations and bias associated with visual scoring of any type [19]. Just as expert breeders, pathologists, and physiologists can disagree among themselves on how to classify subtle phenotypic differences, the CNN developed here notably had a case of consistent disagreement with the visual scores from the expert that were actually used to train it. Indeed, it has been shown in different fields how deep learning can surpass the accuracy of experts [20]. Moving beyond the input of a single person, deep learning for high-throughput phenotyping has the potential power to synthesize the consensus knowledge of an entire community of experts through training on shared datasets. When combined with high-resolution imaging that is becoming more easily acquired from unmanned aerial systems, the collection of this level of image data across many research groups and breeding programs could develop robust training sets for all phenotypes of interest along with the built-in feature of consensus from many experts.

## Potential Implications

The first generation of high-throughput plant phenotyping has focused on sensor and image features that can be directly mapped to plant phenotypes, but it remains limited on exploring the full scope of phenotypic variants. Conceptually, deep learning approaches for the next generation of high-throughput phenotyping can be extended to any trait of interest in any species for which high-resolution imaging and expert scoring of the phenotypes can be obtained. This development in high-throughput plant phenotyping can enable breeders and geneticists to measure complex phenotypes on the size of populations that are used in breeding programs and is needed to understand gene function on a genome-wide scale and uncover genetic variants to develop vastly improved varieties for a future of food security.

## Methods

### Development of field-based high-throughput platform for image collection

A phenotyping mobile unit (“PheMU”) [15] was developed to image winter wheat field plots at Kansas State University, Manhattan, Kansas, USA. PheMU was constructed on a high-clearance sprayer (Bowman Mudmaster, Bowman Manufacturing Co., Inc., Newport, Arkansas, USA) with a height-adjustable sensor boom to capture images throughout the growing season from consistent distance from the canopy. An imaging array including multiple digital single-lens reflex (DSLR) cameras (EOS 7D, Canon, Ohta-ku, Tokyo, Japan) was carried by the PheMU to capture high-resolution crop images. For georeferencing images, 2 GNSS antennae (AG25, Trimble, Sunnyvale, California, USA) were installed at each end of the sensor boom and connected to an RTK GNSS receiver (BX982, Trimble, Sunnyvale, California, USA). A laptop computer was used to control cameras, collect images, and log positioning data. To reduce the shadows on the canopy and capture images in a balanced light condition, a rectangle-shape shade sail (Kookaburra OL0131REC, Awnings-USA, Camanche, Iowa, USA) was mounted over the sensor boom.

### Plant materials and field experiments

Two populations were used in this study, (i) a recombinant inbred line (RIL) population consisting of 318 RILs developed from single seed descent to the F_5_ generation from a cross between US winter wheat varieties “Lakin” and “Fuller,” with seed for field trials increased from a single plant in the F_5_ generation; and (ii) a diverse panel of winter wheat inbred lines (Diversity Panel) consisting of 340 lines (Supplementary Data S1) that was an augmented panel from a previously described set of 299 historical and current winter wheat cultivars [21].

Field trials were planted at the Kansas State University Ashland Bottoms research farm (39 7.015 N, 96 37.003 W) on 10 October 2015 and 18 October 2016 for the Lakin × Fuller and 20 October 2015 and 19 October 2016 for the diversity panel for the 2 years, respectively. Trials were planted in 2 replications of an augmented incomplete design with 1 check plot per block of “Lakin” and “Fuller” for the Lakin × Fuller population and “Everest” for the AM Panel (Supplementary Data S1).

Phenotypic measurements for awns and percent heading were visually scored and recorded using FieldBook [22]. Awn morphology was scored on the diversity panel according to Crop Ontology CO_321:0000027 [23]. The Lakin × Fuller population is completely awned. Percent heading was visually scored at 2–4 day intervals through the season, scored corresponding to developmental stages of Zadoks et al. [17] 49–59 (Crop Ontology CO_321:0000476). Percent heading was scored as the percentage of heads emerged from the boot to give a direct indicator with multiple linear classes from 0% to 100% of heading progression to model with the deep learning. A score of 50% heading was given consistent with the standard visual observation of heading date when 50% of the spike is emerged on 50% of all stems (Crop Ontology CO_321:0000840). For imaging timepoints that corresponded to visual measurements on the same date, we assigned labels to all images from a given plot on that date with the visual score for that respective plot.

For imaging timepoints that did not correspond with visual measurement dates, the successive visual measurements for dates directly before and after the imaging date were used to impute the percentage heading labels for images on that date. A weighted average of the visual scores based on number of days from the imaging date was used as follows:

$$\begin{equation*}
\mathrm{PC}{\mathrm{T}_t} = \left[ {\mathrm{PC}{\mathrm{T}_{t - 1}}*({T_t} - {T_{t - 1}}) + \mathrm{PC}{\mathrm{T}_{t + 1}}*({T_{t + 1}} - {T_t})} \right]*\frac{1}{{({T_{t - 1}} - {T_{t + 1}})}},
\end{equation*}$$

where PCT is the visual score of percentage heading at the corresponding timepoint and *t* is the respective timepoint in days. The timepoints *t*− 1 and *t*+ 1 correspond to the timepoints of the respective visual scores directly before and after the date of imaging. This gives a weighted average of the 2 successive visual measurements for imaging timepoints that did not correspond to the same days when visual measurements were taken.

### Field mapping and image collection

Proximal imaging of the field plots was also conducted at target intervals of 2–3 days. Owing to rain and wet field conditions, there was a gap in imaging date in 2017 of 5 days. Cameras in the imaging array were set to capture proximal images of wheat plots in nadir and off-nadir view angles, and from different parts of each plot. The PheMU was operated at 0.3–0.5 m/s with the cameras positioned at 0.5 m above the canopy. Each DSLR camera was triggered to take mid-size JPEG images (8 megapixels) in 1.25 Hz by a C# program using the Canon EOS Digital SDK (EDSDK v2.14, Canon, Ohta-ku, Tokyo, Japan). To capture unblurry and focused images on a mobile platform, the camera was set using manual focus, 1/500-second shutter speed, and f/5 aperture. The camera ISO was adjustable according to the light condition at the beginning of each image acquisition. Each image was directly transferred from the camera to the laptop computer. Image file names and the time stamps when captured were logged in a text file for subsequent georeferencing. Images were then georeferenced and positioned to individual field plots using the approach of Wang et al. [24]. The boundary coordinates of each field plot were delineated in Quantum GIS (QGIS [25]) using an orthomosaic field map generated from aerial images using the approach of Haghighattalab et al. 2016 [5]. Images inside each plot boundary were geotagged with designated plot IDs to be linked with the plot level scores (as described above) and genotype information based on the entry for that respective field plot (Supplementary Dataset).

### Neural networks

We followed the standard approach of starting with an existing network pre-trained on the Imagenet dataset [26] and fine-tuning that network to give optimal performance for our task. We subsequently made slight modifications to standard CNN to optimize the network for the phenotyping task. For the baseline model we used Resnet [27], which has been previously used for many applications including people re-identification [28] and flower species identification [29].

#### Training data preparation and size

For the awned phenotype, training and validation images were from a 2017 association mapping panel (AM Panel) containing 700 plots, of which 29 plots were awnless and the remaining were awned. As a validation dataset, we reserved 70 plots from the awned and 5 plots from the awnless and sampled images from the remaining plots as the training data. For the training set, we used 2,000 images for awned plots and 1,800 images for awnless plots. The “WheatNet” network was trained with the following parameters: mini-batch stochastic gradient with a batch size of 44. The learning rate is initialized at 0.01 and was reduced by 80% every 5 training epochs. Training continued for 30 epochs.

For estimating heading percentage the training dataset is from the AM-Panel image set from 2016 and 2017, which consists of 711 plots. In these plots, the training set contains 611 randomly sampled plots, and the validation set contains the remaining 100 plots. Each plot was imaged on multiple dates, and on each date, multiple images were taken of each plot. Each plot was assigned 1 of 11 classes, corresponding to a visual score of percentage heading of 0, 10, 20, … 100. To create a training dataset, images from each plot were randomly selected to get 2,000 images per class. A validation dataset was sampled from images from the 100 plots from the diversity panel by randomly selecting 200 images for each class.

Resnet, and most CNNs, are presently restricted in the size of the image they can analyze owing to current hardware limitations (e.g., GPU memory) used for training the networks. We therefore cropped the large images captured by the tractor system into 3 by 3 blocks (patches). Each of the 2,000 images per class give 9 ×2000 patches per class. Therefore, overall we trained the networks with 198,000 image patches cropped from 22,000 images. The test dataset comes from the Lakin × Fuller RIL population in 2017. This dataset contains ∼80,000 images per class, and all images came from 676 plots and germplasm that were never seen in the test or validation data.

To develop a more robust CNN for heading percentage we developed 2 important modifications of the base network.

#### *Modification 1:* Error function that gives partial credit for classifications that are within 10–20% of the label

The output of a CNN can be viewed as a probability distribution of classes, with close percentage classes being more similar. Meanwhile, a given visual labeled maturity percentage can have a ±10% or ±20% offset mislabeled condition. In our data, we evaluated repeatedly visual scoring a small subset of the field plots and estimated the percentage of the mislabeled images that have a discrepancy of 10% at ∼10% in each class and that have a discrepancy of 20% at ∼5% in each class. There was negligible discrepancy of ≥30%. Therefore, the image label was modeled as a distribution that the labeled class has the value 0.7 for correct class, 0.1 for 10% discrepancy, and 0.05 for 20% discrepancy, respectively, and the remaining classes have value 0, which ensure that the sum of all class probability is 1. The error function calculates the average mismatch of the probability of output and the target distribution, which calculate the absolute difference of each class value between output and the target value.

#### *Modification 2:* WheatNet

In order to keep as much of the full details of the images as possible, which is sensitive to maturity classification, the modified architecture keeps the input image with the resolution of 672 × 672 pixels. The main idea of the design of the architecture is to mimic how experienced individuals assign a phenotype (e.g., the maturity percentage) from a wheat plot or image, by taking a consensus from viewing all parts of the images. To capture this feature of visual scoring in the CNN, the network classified the maturity percentage of each image patch and then summarized all predictions and gave output for the consensus prediction for a whole image. The validation dataset for each network was used to determine the optimal number of training epochs and hyper-parameters. Full detail of each layer in the WheatNet is included as Supplementary Table S3.

### Genetic analysis

RILs from the Lakin × Fuller population were genotyped using genotyping-by-sequencing with 2 enzymes, PstI and MspI [30]. Two sets of libraries for the RILs and replicated samples of the parents were made in 95-plexing and 190-plexing and sequenced with Illumina HiSeq2000 and NextSeq, respectively. Single-nucleotide polymorphisms (SNPs) were called using TASSEL 5 GBS v2 pipeline [31] anchored to the IWGSC wheat genome v1.0 assembly [32] with the following parameters: -mnQS 10, enzyme PstI-MspI, -c 20, -minMAPQ 20, and -mnMAF 0.1. Unique sequence tags were mapped to the wheat reference genome (Chinese Spring) using bowtie2 [33] with the following settings: –end-to-end -D 20 -R 3 -N 0 -L 10 -i S,1,0.25. SNPs passing ≥1 criterion were recovered: inbreeding coefficient of ≥0.8, Fisher exact test (*P* < 0.001) to determine bi-allelic single locus SNPs [30], and χ^2^ test for bi-allelic segregation with 96% expected inbreeding. SNPs having 2 parents homozygous within and polymorphic between were extracted and missing loci were imputed with LB-impute [34] with parameter settings of -readerr 0.1 -genotypeerr 0.1 -window 7. Finally, SNP sites were removed if minor allele frequency (MAF) < 0.1, missing > 30%, or heterozygosity > 6%. The TASSEL pipeline gave ∼82% useable reads out of 2.15 billion reads. The overall alignment of 1,973,081 unique tags against the reference genome was 91%, with a unique alignment of 37.8%. A total of 44,679 SNPs was discovered, of which 28,972 passed filtering. We then filtered RILs for missing data and heterozygosity, resulting in 306 RILs and the 2 parents with suitable genotypes. Finally, 8,797 SNPs were recovered after imputation with the additional filtering for MAF, missing, and heterozygosity. All raw sequencing data for the Lakin × Fuller RIL population are available from the NCBI SRA under accession number SRP136362.

Using traits directly from visual scoring and from the network image classification by the K-net we calculated trait distributions, variance components, and best linear unbiased predictors in R statistical software [35] (Supplemental Information).

To model heading date from time-series scoring/predictions of percent heading, we fit a logistic growth curve model for each individual plot according to the function

$$\begin{equation*}
{y_i} = \frac{{\mathrm{phi}1}}{{1 + {e^{ - \left( {\mathrm{phi}2\,\,+\,\, \mathrm{phi}3\,\,*\,\,\mathrm{day}} \right)}}}}\ ,
\end{equation*}$$

where ${y_i}$ is the *i*^th^ observation of heading percentage for a given plot. phi1 is the asymptote maximum and was set to fixed at 100 for maximum percent heading. This model allows for different rates of development through heading as defined by phi2 and phi3. The independent variable "day" was calculated as the day of the year for observation *i*. The model was fit using the nls function from the nlme package [36]. To increase the robustness of fit we added points of 0 and 100% heading at 10, 20, and 30 days before the first and after the last visual measurements, respectively, corresponding to dates when all of the plots were not started and completely headed. The heading date for each plot was calculated as the date closest to 50% using the predict function in R at 0.1-day increments over the full range of the days.

For heading date, phi2 and phi3, we calculated broad-sense heritability on a line mean basis according to Holland et al. [37] for replicated trials of inbred lines (e.g., clonal species) in 1 location within 1 year as:

$$\begin{equation*}
{H^2} = {\frac{\sigma _G^2} {\sigma _G^2 + \frac{{\sigma _e^2}}{r}}},\
\end{equation*}$$

where $\sigma _G^2$ is the total genetic variance for entries in the trial, $\sigma _e^2$ is the error variance, and *r* is the number of replications. Heritability can be estimated across multiple years at 1 location as follows:

$$\begin{equation*}
{H^2} = {\frac{\sigma _G^2}{\sigma _G^2 + \frac{{\sigma _{\mathrm{GY}}^2}}{y} + \frac{{\sigma _e^2}}{{yr}}}},\
\end{equation*}$$

where $\sigma _{GY}^2$ is the genotype by year variance and *y* is the number of years evaluated. Variance components were estimated by fitting mixed models in the asreml package [38] in R. Models were fit with random effects for entry, year, and replication within year and using a row-column autoregressive variance structure using the following model:

$$\begin{equation*}
{y_{ijk}} = {g_i}\ + {y_j} + {r_{k\left( j \right)}} + e,
\end{equation*}$$

where ${y_{ijk}}$ is the observed plot-level phenotype, ${g_i}$ is the random-effect genotype effect of entry *i* distributed as independent and identically distributed random variables where ${g_i}\sim \ N( {0,\ \sigma _i^2} )$, ${y_j}$ is the random effect for year *j*, ${r_{k( j )}}$ is the random effect of replication *k* within year *j*, and *e* is the residual variance partitioned with a 2-dimensional autoregressive spatial structure (AR1 ⊗ AR1). Best linear unbiased estimates were estimated for each entry within and across years by fitting the same model with entry as a fixed effect and using the predict function in R.

Following calculation of heading date, we observed a bimodal distribution of heading dates from the multi-year model BLUPs (Supplemental Fig. S4). The distribution was delimited at 124 days to calculate the number of “early” and “late” lines. The number of lines in each group was fit to a ${\chi ^2}$ test for 2 classes with probability of 0.75 and 0.25 according to a 2-gene dominant epistasis model for inbred lines using the chisq.test function in R.

We tested for genetic association of heading date in the Lakin × Fuller population as measured by the logistic regression using a standard mixed model:

$$\begin{equation*}
y\ = \ Wv + X\beta + Zu + e,
\end{equation*}$$

where *y* is the projected phenotype of heading date (50% intersect) or rate of heading (phi3) from the logisitic regression models. The use of a bi-parental population without population structure or kinship greatly simplified the equation to

$$\begin{equation*}
{y_i} = {\beta _{i,k}}\ + e,
\end{equation*}$$

where ${\beta _{i,\ k}}$ is the allele substitution effect for locus *k* in individual *i*, and *e* is residual error. Each marker effect was estimated using the lmer function in R and Bonferroni for multiple testing correction of experimental α of 0.05.

Following identification of significant marker association, we tested for 2-way epistatic interactions for all markers that were associated with heading date using the following model:

$$\begin{equation*}
{y_i} = {\alpha _{i,j}} + {\beta _{i,k}} + {\varepsilon _{i,j \cdot k}} + e,
\end{equation*}$$

where ${y_i}$is the phenotype of the individual *i*; ${\alpha _{i,\ j}}$ is the allele substitution effect for locus *j* in individual *i*; ${\beta _{i,\ k}}$ is the allele substitution effect for locus *k* in individual *i*; ${\varepsilon _{i,j \cdot k}}$ is the interaction between locus *j* and *k*; and *e* is residual error.

## Availability of Source Code and Requirements

- Availability of source code: Code used for camera control is available at [39]. Code used for neural networks is available at [40]. Code used for genetic analysis is available at [41].
- Operating system(s): Program used for camera control is run on Windows 10 operating system. Other programs are platform independent.
- Programming language: Code used for camera control is scripted in C#. Code used for neural networks is scripted in Python. Code used for genetic analysis is scripted in R.
- Other requirements: The code for deep learning is run on the Pytorch (Version 0.3). The computer used for deep learning required ≥2 GPUs with 12 GB memory on each one.
- License: R as a package is licensed under GNU GPL.

## Availability of Supporting Data and Materials

Snapshots of our code and other supporting data are openly available in the GigaScience repository [16].

## Additional files

**Figure S1**. Example of geolocations of images from one camera (yellow points) marked on the field map (white polygons on the orthomosaic photo). GIS datasets (shape files) for all positioned images are included as supplemental data.

**Figure S2**. Example of (a) awnless and (b) awned phenotypes from the image dataset for the diversity panel. (c and d) Image examples of misclassified entry “MFA-2018” showing a heterogenous atypical awnlette phenotype.

**Figure S3**. The confusion matrix for (a) training, (b) validation, and (c) testing the CNN for predicting percentage heading.

**Figure S4**. Phenotypic distribution of Lakin × Fuller recombinant inbred lines for heading date (day of year) in 2017 field trial.

**Figure S5**. Genome-wide association testing of phi2 (slope of logistic regression). No significant markers above Bonferroni multiple-test correction threshold.

**Table S1**. Individual image predictions (a) and accuracy (b) for awns phenotypes.

**Table S2**. Supplemental dataset table of field plot entries and field design for Diversity Panel and Lakin × Fuller Recombinant Inbred Line (RIL) populations in 2016 and 2017.

**Supplemental Table S3**. Full details of each layer in the WheatNet.

**Table S4**. Analysis of variance for full interaction model of *Ppd-D1, Ppd-B1*, and locus on 1B. test = lm(Y ∼ g.2B * g.2D * g.1B).

giz120_GIGA-D-18-00431_Original_Submission

giz120_GIGA-D-18-00431_Revision_1

giz120_GIGA-D-18-00431_Revision_2

giz120_Response_to_Reviewer_Comments_Original_Submission

giz120_Response_to_Reviewer_Comments_Revision_1

giz120_Reviewer_1_Report_Original_Submission Valerio Giuffrida -- 2/7/2019 Reviewed

giz120_Reviewer_1_Report_Revision_1 Valerio Giuffrida -- 5/31/2019 Reviewed

giz120_Reviewer_2_Report_Original_Submission Ian Stavness, Ph.D. -- 2/24/2019 Reviewed

giz120_Supplemental_Files

## Abbreviations

AM Panel: association mapping panel; BLUP: best linear unbiased prediction; CNN: convolutional neural network; DSLR: digital single-lens reflex; GPU: graphics processing unit; HTP: high-throughput phenotyping; NCBI: National Center for Biotechnology Information; PheMU: phenotyping mobile unit; QTL: quantitative trait locus; RIL: recombinant inbred line; SNP: single-nucleotide polymorphism; SRA: Sequence Read Archive.

## Competing interests

The authors declare no competing interests. The plant materials tested in this study are public germplasm and/or were tested in accordance with local, national, and international guidelines and legislation with the appropriate permissions and/or licenses for the present study.

## Funding

This work was supported by the National Science Foundation (NSF) Plant Genome Research Program (PGRP) (Grant No. IOS-1238187), the Kansas Wheat Commission and Kansas Wheat Alliance, the US Agency for International Development (USAID) Feed the Future Innovation Lab for Applied Wheat Genomics (Cooperative Agreement No. AID-OAA-A-13-00051), and by the NIFA International Wheat Yield Partnership (Grant No. 2017-67007-25933/project accession no. 1011391) from the USDA National Institute of Food and Agriculture. The opinions expressed herein are those of the author(s) and do not necessarily reflect the views of the US Agency for International Development, the US National Science Foundation, or the US Department of Agriculture. The funders had no role in study design, data collection, or analysis.

## Authors' contributions

J.P. conceived and designed the study. B.E. managed the field trials and collected phenotypic data. X.W. developed the phenotyping platform and collected all image data. H.X. and R.P. analyzed images and developed neural networks. S.S. analyzed genetic data. J.P. directed the overall project and analyzed genetic and phenotypic data. J.P., X.W., H.X., and R.P. wrote the manuscript. All authors reviewed and approved the manuscript.

## Acknowledgements

We sincerely appreciate the assistance of Shuangye Wu in genotyping, Mark Lucas and Josiah Altschuler in data curation, and Haley Ahlers in graphics design, along with all members of the Wheat Genetics Lab at Kansas State University for project support, input, and feedback.

## References

[1] Tester M, Langridge P Breeding technologies to increase crop production in a changing world. Science. 2010;327(5967):818–22. 20150489 10.1126/science.1183700

[2] Furbank RT, Tester M Phenomics – technologies to relieve the phenotyping bottleneck. Trends Plant Sci. 2011;16(12):635–44. 22074787 10.1016/j.tplants.2011.09.005

[3] Andrade-Sanchez P, Gore MA, Heun JT, et al. Development and evaluation of a field-based high-throughput phenotyping platform. Functional Plant Biol. 2014;41(1):68–79. 10.1071/FP13126 32480967

[4] Pauli D, Andrade-Sanchez P, Carmo-Silva AE, et al. Field-based high-throughput plant phenotyping reveals the temporal patterns of quantitative trait loci associated with stress-responsive traits in cotton. G3 (Bethesda). 2016;6(4):865–79. 26818078 10.1534/g3.115.023515 PMC4825657

[5] Haghighattalab A, González Pérez L, Mondal S, et al. Application of unmanned aerial systems for high throughput phenotyping of large wheat breeding nurseries. Plant Methods. 2016;12(1):1–15. 27347001 10.1186/s13007-016-0134-6 PMC4921008

[6] LeCun Y, Bengio Y, Hinton G Deep learning. Nature. 2015;521(7553):436. 26017442 10.1038/nature14539

[7] Krizhevsky A, Sutskever I, Hinton GE ImageNet classification with deep convolutional neural networks. In: Advances in Neural Information Processing Systems. 2012:1097–105.

[8] Ubbens JR, Stavness I Deep plant phenomics: A deep learning platform for complex plant phenotyping tasks. Front Plant Sci. 2017;8:1190. 28736569 10.3389/fpls.2017.01190 PMC5500639

[9] Pound MP, Atkinson JA, Townsend AJ, et al. Deep machine learning provides state-of-the-art performance in image-based plant phenotyping. Gigascience. 2017;6(10):1–10. 10.1093/gigascience/gix083 PMC5632296 29020747

[10] Dobrescu A, Valerio Giuffrida M, Tsaftaris SA Leveraging multiple datasets for deep leaf countingIn: Proceedings of the IEEE International Conference on Computer Vision. 2017:2072–2079.

[11] Giuffrida MV, Doerner P, Tsaftaris SA Pheno‐deep counter: A unified and versatile deep learning architecture for leaf counting. Plant J. 2018;96(4):880–90. 30101442 10.1111/tpj.14064 PMC6282617

[12] Ghosal S, Blystone D, Singh AK, et al. An explainable deep machine vision framework for plant stress phenotyping. Proc Natl Acad Sci U S A. 2018;115(18):4613. 29666265 10.1073/pnas.1716999115 PMC5939070

[13] Uzal LC, Grinblat GL, Namías R, et al. Seed-per-pod estimation for plant breeding using deep learning. Comput Electron Agric. 2018;150:196–204.

[14] Hasan MM, Chopin JP, Laga H, et al. Detection and analysis of wheat spikes using convolutional neural networks. Plant Methods. 2018;14(1):100. 30459822 10.1186/s13007-018-0366-8 PMC6236889

[15] Barker J, Zhang N, Sharon J, et al. Development of a field-based high-throughput mobile phenotyping platform. Comput Electron Agric. 2016;122:74–85.

[16] Wang X, Xuan H, Evers B, et al. Supporting data for “High throughput phenotyping with deep learning gives insight into the genetic architecture of flowering time in wheat.”. GigaScience Database. 2019 10.5524/100566. PMC6862935 31742599

[17] Zadoks JC, Chang TT, Konzak CF A decimal code for the growth stages of cereals. Weed Res. 1974;14(6):415–21.

[18] DeChant C, Wiesner-Hanks T, Chen S, et al. Automated identification of northern leaf blight-infected maize plants from field imagery using deep learning. Phytopathology. 2017;107(11):1426–32. 28653579 10.1094/PHYTO-11-16-0417-R

[19] Poland J, Nelson R In the eye of the beholder: The effect of rater variability and different rating scales on QTL mapping. Phytopathology. 2011;101(2):290–8. 20955083 10.1094/PHYTO-03-10-0087

[20] Han SS, Park GH, Lim W, et al. Deep neural networks show an equivalent and often superior performance to dermatologists in onychomycosis diagnosis: Automatic construction of onychomycosis datasets by region-based convolutional deep neural network. PLoS One. 2018;13(1):e0191493. 29352285 10.1371/journal.pone.0191493 PMC5774804

[21] Grogan SM, Anderson J, Baenziger PS, et al. Phenotypic plasticity of winter wheat heading date and grain yield across the us great plains. Crop Sci. 2016;56(5):2223–36.

[22] Rife TW, Poland JA Field book: An open-source application for field data collection on android. Crop Sci. 2014;54(4):1624–7.

[23] Crop Ontology Curation Tool. www.cropontology.org. Accessed on 31 December 2011.

[24] Wang X, Thorp KR, White JW, et al. Approaches for geospatial processing of field-based high-throughput plant phenomics data from ground vehicle platforms. Trans ASABE. 2016;59(5):1053.

[25] QGIS: A Free and Open Source Geographic Information System. www.qgis.org.

[26] Deng J, Dong W, Socher R, et al. Imagenet: A large-scale hierarchical image database. In: 2009 IEEE Conference on Computer Vision and Pattern Recognition, Miami, FL, USA. IEEE; 2009:248–55.

[27] He K, Zhang X, Ren S, et al. Deep residual learning for image recognition. In: 2016 IEEE Conference on Computer Vision and Pattern Recognition (CVPR), Las Vegas, NV, USA. IEEE; 2016:770–8.

[28] Hermans A, Beyer L, Leibe B In defense of the triplet loss for person re-identification. arXiv. 2017:170307737.

[29] Nguyen TTN, Le VT, Le TL, et al. Flower species identification using deep convolutional neural networks. In: AUN/SEED-Net Regional Conference for Computer and Information Engineering. 2016.

[30] Poland JA, Brown PJ, Sorrells ME, et al. Development of high-density genetic maps for barley and wheat using a novel two-enzyme genotyping-by-sequencing approach. PLoS One. 2012;7(2):e32253. 22389690 10.1371/journal.pone.0032253 PMC3289635

[31] Glaubitz JC, Casstevens TM, Lu F, et al. Tassel-gbs: A high capacity genotyping by sequencing analysis pipeline. PLoS One. 2014;9(2):e90346. 24587335 10.1371/journal.pone.0090346 PMC3938676

[32] The genome assembly of Triticum aestivum cv. Chinese Spring. https://wheat-urgi.versailles.inra.fr/Seq-Repository/Assemblies. Accessed on 3 September 2013.

[33] Langmead B, Trapnell C, Pop M, et al. Ultrafast and memory-efficient alignment of short DNA sequences to the human genome. Genome Biol. 2009;10(3):1–10. 10.1186/gb-2009-10-3-r25 PMC2690996 19261174

[34] Fragoso CA, Heffelfinger C, Zhao H, et al. Imputing genotypes in biallelic populations from low-coverage sequence data. Genetics. 2016;202(2):487. 26715670 10.1534/genetics.115.182071 PMC4788230

[35] R Core Team. R: A language and environment for statistical computing. Vienna, Austria: R Foundation for Statistical Computing; 2016.

[36] Pinheiro J, Bates D, DebRoy S, et al. Nlme: Linear and nonlinear mixed effects models. R package version 3.1-131 ed 2017.

[37] Holland J, Nyquist W, Cervantes-Martinez C Estimating and interpreting heritability for plant breeding: An update. In:

[38] Butler D Asreml: Asreml() fits the linear mixed model. R package version 3.0. ed 2009.

[39] The camera control program. https://github.com/xwangksu/CamControl. Accessed on 19 April 2018.

[40] The neural networks program. https://github.com/littleredxh/WheatNet. Accessed on 6 December 2018.

[41] The genetics analysis program. https://github.com/jessepoland/htp-analysis. Accessed on 21 January 2019.

