# Supplementary material for: High-throughput phenotyping with deep learning gives insight into the genetic architecture of flowering time in wheat
Source: Gigascience. 2019 Nov 19;8(11):giz120. doi: 10.1093/gigascience/giz120 (PMC6862935; doi:10.1093/gigascience/giz120)
Supplement: giz120_GIGA-D-18-00431_Revision_1 [file giz120_giga-d-18-00431_revision_1.pdf]

GigaScience

High-throughput phenotyping with deep learning gives insight into the genetic architecture of flowering time in wheat

--Manuscript Draft--

**Manuscript Number:**	GIGA-D-18-00431R1
**Full Title:**	High-throughput phenotyping with deep learning gives insight into the genetic architecture of flowering time in wheat
**Article Type:**	Research
**Funding Information:**	Directorate for Biological Sciences (IOS-1238187) — Dr Jesse Poland<br>United States Agency for International Development (AID-OAA-A-13-00051) — Dr Jesse Poland<br>National Institute of Food and Agriculture (Grant No. 2017-67007-25933/project accession no. 1011391) — Dr Jesse Poland
**Abstract:**	Background: Precise measurement of plant traits with precision and speed on large populations has emerged as a critical bottleneck in connecting genotype to phenotype in genetics and breeding. This bottleneck limits advancements in understanding plant genomes and the development of improved, high-yielding crop varieties.<br><br>Results: Here we demonstrate the application of deep learning on proximal imaging from a mobile field vehicle to directly estimate plant morphology and developmental stages in wheat under field conditions. We developed and trained a convolutional neural network with image datasets labeled from expert visual scores and used this 'breeder-trained' network to classify wheat morphology and developmental stages. For both morphological (awned) and phenological (flowering time) traits, we demonstrate high heritability and extremely high accuracy against the 'ground-truth' values from visual scoring. Using the traits predicted by the network, we tested genotype-to-phenotype association using the deep learning phenotypes and uncovered novel epistatic interactions for flowering time. Enabled by the time-series high-throughput phenotyping, we describe a new phenotype as the rate of flowering and show heritable genetic control for this trait.<br><br>Conclusions: We demonstrated a field-based high-throughput phenotyping approach using deep learning that can directly measure morphological and developmental phenotypes in genetic populations. Most powerfully, the deep learning approach presented here gives a conceptual advancement in high-throughput plant phenotyping as it can potentially estimate any trait in any plant species through leveraging expert knowledge from breeders, geneticist, pathologists and physiologists.
**Corresponding Author:**	Jesse Poland<br>Kansas State University<br>Manhattan, Kansas UNITED STATES
**Corresponding Author Secondary Information:**	
**Corresponding Author's Institution:**	Kansas State University
**Corresponding Author's Secondary Institution:**	
**First Author:**	Xu Wang
**First Author Secondary Information:**	
**Order of Authors:**	Xu Wang<br>Hong Xuan<br>Byron Evers

	Sandesh Shrestha
	Robert Plesse
	Jesse Poland
**Order of Authors Secondary Information:**	
**Response to Reviewers:**	Review response and manuscript with tracked changes included as supplemental documents
**Additional Information:**	
**Question**	**Response**
Are you submitting this manuscript to a special series or article collection?	No
**Experimental design and statistics**<br><br>Full details of the experimental design and statistical methods used should be given in the Methods section, as detailed in our Minimum Standards Reporting Checklist. Information essential to interpreting the data presented should be made available in the figure legends.<br><br>Have you included all the information requested in your manuscript?	Yes
**Resources**<br><br>A description of all resources used, including antibodies, cell lines, animals and software tools, with enough information to allow them to be uniquely identified, should be included in the Methods section. Authors are strongly encouraged to cite Research Resource Identifiers (RRIDs) for antibodies, model organisms and tools, where possible.<br><br>Have you included the information requested as detailed in our Minimum Standards Reporting Checklist?	Yes
**Availability of data and materials**<br><br>All datasets and code on which the conclusions of the paper rely must be either included in your submission or	Yes

deposited in publicly available repositories (where available and ethically appropriate), referencing such data using a unique identifier in the references and in the "Availability of Data and Materials" section of your manuscript.<br><br>Have you have met the above requirement as detailed in our Minimum Standards Reporting Checklist?	

Manuscript
Click here to access/download;Manuscript;Manuscript-GigaScience_DeepWheat_REVISION-20190524-clean.pdf
Click here to view linked References

High-throughput phenotyping with deep learning gives insight into the genetic architecture of flowering time in wheat

Xu Wang[1,a], Hong Xuan[2,a], Byron Evers[1], Sandesh Shrestha[1], Robert Pless[2,*], and Jesse Poland[1,a,*]

[1] Department of Plant Pathology, Kansas State University, Manhattan, KS 66506
[2] Department of Computer Science, George Washington University, Washington D.C.
[a] These authors contributed equally to this work

*Corresponding Authors:
Robert Pless (pless@gwu.edu)
Jesse Poland (jpoland@ksu.edu)

ABSTRACT

Background: Precise measurement of plant traits with precision and speed on large populations has emerged as a critical bottleneck in connecting genotype to phenotype in genetics and breeding. This bottleneck limits advancements in understanding plant genomes and the development of improved, high-yielding crop varieties.

Results: Here we demonstrate the application of deep learning on proximal imaging from a mobile field vehicle to directly estimate plant morphology and developmental stages in wheat under field conditions. We developed and trained a convolutional neural network with image datasets labeled from expert visual scores and used this 'breeder-trained' network to classify wheat morphology and developmental stages. For both morphological (awned) and phenological (flowering time) traits, we demonstrate high heritability and extremely high accuracy against the 'ground-truth' values from visual scoring. Using the traits predicted by the network, we tested genotype-to-phenotype association using the deep learning phenotypes and uncovered novel epistatic interactions for flowering time. Enabled by the time-series high-throughput phenotyping, we describe a new phenotype as the rate of flowering and show heritable genetic control for this trait.

Conclusions: We demonstrated a field-based high-throughput phenotyping approach using deep learning that can directly measure morphological and developmental phenotypes in genetic populations. Most powerfully, the deep learning approach presented here gives a conceptual advancement in high-throughput plant phenotyping as it can potentially estimate any trait in any plant species through leveraging expert knowledge from breeders, geneticist, pathologists and physiologists.

Keywords:

convolutional neural network, deep learning, genetic architecture, plant breeding, wheat

BACKGROUND

Limitations in phenotyping are widely recognized as a critical constraint in genetic studies and in plant breeding [1, 2]. Initial developments in field-based, high-throughput phenotyping (HTP) have focused on direct sensor or image measurements to extract proxies for traits of interest such as vegetation indexes from spectral reflectance [3, 4] or plant height from digital elevation models [5]. While lending great insight to plant processes, this first-generation of HTP is limited in assessment of 'complex' traits such as plant morphology or growth stage that that cannot be assessed by a linear function of pixel values. While these complex morphological and developmental features are readily distinguished by a trained eye, the assessment of these phenotypes with high-throughput platforms is challenging, particularly under field conditions used in plant breeding programs.

Deep learning has emerged as a powerful machine learning approach that takes advantage of both the extraordinary computing power and very large datasets that are often now available [6]. Deep learning bypasses the need to explicitly define which features are most useful or needed for data analysis. Instead deep learning discovers a complete end-to-end process to map data samples to outputs that are consistent with the large, labelled datasets used for training the network. For image analysis tasks, convolutional neural networks (CNNs) learn this end-to-end mapping by optimizing for many layers of filters. The first filters are easily interpreted as image features (e.g. detecting edges, bright points or color variations), and subsequent layers are increasingly complicated combinations of earlier features. When there is sufficient training data, CNNs dramatically outperform all alternative existing methods for image analysis. For benchmark classification tasks attempting to label which of 1,000 different objects are in an image, results have increased from 84.6% in 2012 to 96.4% in 2015 [7].

Based on this impressive performance of the latest CNNs, these deep learning approaches are being applied to challenging tasks in plant phenomics [8] including root and shoot feature identification [9], leaf counting [10, 11], classifying types of biotic and abiotic stress [12], counting seeds per pod [13], and detection of wheat spikes [14]. Initial studies have shown a

wide array of potential applications for CNNs in plant phenomics. With increasingly robust image datasets, the use of CNNs has great potential for accurate estimation of plant phenotypes directly from images.

One challenge that has emerged in using CNNs for plant phenomics is the development of suitable datasets that are sufficiently annotated for training the networks [8, 9]. The labeling of images, particularly when going from weak to stronger annotations, is a time and resource intensive constraint for the future of plant phenomics [10]. While it is a greater challenge to train networks with weaker annotations, the size of these datasets can be greatly expanded beyond what is tractable with extensive manual annotation. Furthermore, approaches that can generate a large number of images with imputed labels from visual scoring as demonstrated in this study have potential to exponentially increase the size and scope of labeled image datasets for the phenomics community.

Given the broad applications and demonstrated success of deep learning, we hypothesized that this deep learning approach could be a powerful tool for estimating phenotypes of interest directly from images in segregating plant populations under field conditions used by breeding programs. Such implementations of HTP would have direct application to improve the efficiency of plant breeding while being directly relevant to the phenotypes of interest to breeders and the sizes of populations utilized these breeding programs. When applied in a relevant context at the scale of entire breeding programs (e.g. thousands to tens-of-thousands of field plots), these phenomics tools can contributed to accelerated development of high-yielding, climate-resilient new varieties.

DATA DESCRIPTION

Field-based high-throughput imaging of wheat plots

To advance high-throughput phenotyping of complex morphological and developmental traits under field conditions, we developed a high-clearance field vehicle [15] equipped with an array of DSLR cameras collecting geo-positioned images (**Figures 1a & 1b**). This platform was

deployed across wheat field trials in 2016 and 2017. Each year we grew two trials, 1) a recombinant inbred line (RIL) population from a cross between wheat cultivars 'Lakin' and 'Fuller', and 2) a panel of diverse historical and modern winter wheat varieties consisting of a total of 1398 plots each year. We captured over 400,000 proximal images of the wheat canopies throughout the growing seasons in 2016 and 2017. These images were geo-referenced and 135,771 and 139,752 of the images were assigned to individual field plots in 2016 and 2017, respectively, based on surveyed coordinates of the field plots and geo-tagged images (**Fig. 1, Supplementary Fig. S1**). This approach enabled high-throughput proximal imaging on an individual plot level (1.5m x 2.4m plot size). Concordant with imaging, field plots were visually scored for percent heading and spike morphology of awned or awnless. To generate a large collection of labeled images suitable for deep learning while avoiding time consuming manual annotations, the images from a given plot were labeled with the 'breeder scores' of awned/awnless and percentage heading visually assessed at the same time points on the same respective plots as the image data collection (**Fig. 1c**). The labeled image data set(s) collected and analyzed in this study are available in the *Giga*DB repository, [persistent identifier].

ANALYSIS

Development of convolutional neural networks

To assess plant features that cannot be measured directly by sensors with the high-throughput platform, we developed a CNN network that could be trained using these geo-positioned images that are labeled with visual scores and subsequently automatically classify and estimated the phenotypes of interest. As a starting point, we first approached the qualitative trait of awn morphology (**Supplementary Fig. S2**).

An initial challenge in the development of the CNN was memory constraints that limit the networks to analyzing relatively small images, but the images were captured at very high resolution. Because the relevant image features are quite small (e.g. wheat awns at 1-2mm

width) reducing the size of the image would make these features invisible. We therefore, cropped the images into a three by three grid of nine patches of 224 x 224 pixel size. To build the full 'WheatNet', we then extended the CNN architecture that analyzes images with a small additional network that combined features from the nine patches to create a consensus estimate for the image (**Fig. 2**).

We used this developed CNN architecture in a training-validation-testing approach to predict the awn phenotype in the diverse panel of inbred lines in which there were awned and awnless variants. The training and validation images were from this diversity panel evaluated in 2017 with 700 plots, of which 29 plots were awnless and 671 were awned. Model training used 2000 images for awned plots and 1800 images for awnless plots. As a validation dataset, we sampled 70 plots from the awned and 5 plots from the awnless and left the remaining plots as the training data. We validated the WheatNet on a set of 300 images each from awned and awnless plots. On this set, the network classification matched the visual score at 99.2% on the training set and 98.6% on the validation set.

To test the WheatNet for predicting awn morphology, we applied the network trained with data from 2017 to test images from field trials of the diversity panel in 2016 which contained 12,504 images from 675 awned plots and 32 awnless plots. At the level of individual images, the prediction accuracy was 98.9% for awned and 98.7% for awnless phenotypes (**Supplemental Table S1**). As many images were captured for each plot, we applied a plot-level consensus voting which increased the accuracy to 99.7% for awned and 100% for awnless. Strikingly, we observed that only two plots were inconsistent between visual scoring and the CNN predictions and that these two plots were the same variety ('MFA-2018') across both field replications. Further inspection showed that this variety was a heterogeneous 'atypical awnlette' phenotype (**Supplementary Fig. S2**), indicating that the CNN was able to detect subtle atypical phenotypes that were lost or ignored in the human scoring.

Measurement of percentage heading

Observing initial proof of concept for using deep learning to score a simple morphological trait with two classes, we extended this approach to a more complex problem of developmental phenotypes using time-series imaging. Flowering time is a critical trait under intense selection in natural populations and breeding programs. Due to tightly closed flowers in wheat, spike emergence (heading time or heading date) is used as a close proxy for flowering time in breeding and genetics studies. To estimate the heading date of wheat, which is classically defined as the date in which spikes (ears) have emerged from 50% of the tillers [16], we applied the CNN to classify percentage heading over the longitudinal image datasets. For the initial trait estimation of percent heading we used the same CNN architecture and training approach as was used for predicting the wheat awn phenotype and trained the network with two years of image data from the diversity panel. An additional feature of predicting percentage heading makes this problem different from standard classification problems, in that the breeder labels are given in 10% increments, and there is some inconsistency in these labels. To address this, we modified the algorithm that trains the CNN to give partial credit for predictions that are within 10% and 20% from the assigned label.

On the classification problem for percent heading the network prediction exactly matched the ten percentile classifications from visual assessment on 45.12% (training set) and 41.27% (validation set) of the images, which is much better than a random guess at 9.10% (1 out of 11 classes). The confusion matrix for training, validation and testing the CNN for predicting percent heading showed clear diagonal patterns indicting linear consistency between the observed and predicted values (**Supplemental Figure S3**). Though there was lower accuracy in the testing phase, the diagonal linear pattern remained consistent. From these observations, we had strong evidence that the CNN is accurately estimating percentage heading for images across the range of heading values and throughout the season. Following this conclusion, we applied the CNN trained on the diversity panel to predict percentage heading and calculate heading date for a biparental RIL population and determine if genotype-to-phenotype associations could be detected directly from phenotypes estimated through deep learning.

To translate the time-series imaging and CNN phenotypic predictions into a single time point for heading date, we applied a logistic regression to the percent heading measurements for each individual plot (**Fig. 3**). From the logistic regression fit, we then found the date intersecting the regression at 50% and assigned this time point as the heading date commensurate with the classical definition of 50% of heads emerged from the boot. Applying this logistic regression individually to each plot we obtained heading dates from the CNN predictions that were highly accurate with the heading date measured directly from visual scoring (**Fig. 4**). We observed over 57% and 88% of heading date measurement were within one day and two days, respectively, with a mean absolute error of 0.99 and mean root square error of 1.25 days. Slope of the regression between the visual and CNN measurements of 1.02 indicated lack of bias from the CNN predictions. Reflecting accurate phenotypes under strong genetic control, the broad-sense heritability for heading date was very high when measured by both visual scoring (H^2=0.982) and CNN predictions (H^2=0.987).

An interesting novel phenotype that can be assessed with this time-series approach is the developmental rate of flowering (heading) progression within an inbred line. This measure of the rate of heading is derived from the slope of the logistic regression. Measuring the slope for each inbred line, we found heritable genetic variance for the rate of heading (H^2_{VISUAL} =0.621, H^2_{CNN}=0.514), indicating that this developmental rate phenotype is also under genetic control. As the rate of heading might simply be an artifact from the heading date *per se*, we tested the correlation between timing and rate of heading and found weak negative correlation (r = -0.19, *p-value* < 0.001). Looking at RILs within the normal early range of heading date (e.g. before May 5th; Day 125) we found no significant correlation (r = 0.078, *p-value* = 0.079) suggesting that the rate of heading is indeed under independent genetic control from heading date.

Genetic analysis of flowering time

Following the measurement of heading date using the neural network, we sought to determine the utility of phenotypes estimated directly from deep learning to uncover the genetic basis of the variation in flowering time present in the biparental population. Though the parental lines

'Lakin' and 'Fuller' are very similar in heading, the progeny showed vast transgressive segregation and also segregation distortion, indicating some underlying epistatic gene action (**Supplementary Fig. S4**). We implemented a genome-wide scan of 8237 genotyping-by-sequencing markers and found strong associations for *PpD-D1* and *PpD-B1* as well as associations on 5B and a novel QTL positioned on at the distal end of chromosome 1B (**Fig. 5**). Suspecting epistatic gene action based on the phenotypic distribution, we tested all significant markers for putative epistatic interactions and found strong interactions between *PpD-D1* and *Ppd-B1* as well as between *PpD-D1* and the locus on 1B (**Fig. 5**). Interestingly, the modeling of the interactions between all three loci removed the main effect of the 1B locus *per se*, with this locus having an opposite effect in the presence of *PpD-D1* early (insensitive) allele (**Fig. 6**).

While we found heritable genetic variance for the rate of heading, we were not able to find any genetic association within this population (**Supplementary Fig. S5**). Implicating missing heritability for the rate of flowering suggested a diffuse genetic architecture of many small effect alleles. We therefore tested whole-genome polygenic models (BayesA and G-BLUP) to capture the genetic variance for the rate of heading. We ran 100 replications of cross-validation predicting 10% masked phenotypes and were able to model 18 to 25% of the heritable genetic variance.

DISCUSSION

From this study, we have demonstrated that deep learning with 'breeder-trained' neural networks from proximal field-based imaging can accurately classify plant morphology. When applied to time-series image datasets this approach can likewise accurately predict developmental stages such as flowering time. Furthermore, these machine vision phenotypes can be used directly to uncover genetic determinants in the populations, connecting genotype to phenotype in the same way as classical approaches to phenotyping.

A significant advancement of the approach presented here is that there is no additional time investment in developing the labeled image set for training. Many applications applying deep learning for image based phenotyping require extensive annotation of the training image sets, such as annotating plant features of interest prior to network training [9, 17]. As demonstrated in this study, HTP image datasets from field trials can be labeled through direct imputation of visual scores routinely collect in the field by breeders. This approach can be used to develop a very large labeled image datasets for training networks on any phenotype of interest without any further human input.

Though the images are labeled, and hence the network subsequently trained, by experienced individuals, there are inherent limitations and bias associated with visual scoring of any type [18]. Just as expert breeders, pathologists and physiologists can disagree among themselves on how to classify subtle phenotypic differences, the CNN developed here notably had a case of consistent disagreement with the visual scores from the expert that was actually used to train it. Indeed, it has been shown in different fields how deep learning can surpass the accuracy of experts [19]. Moving beyond the input of a single person, deep learning for high-throughput phenotyping has the potential power to synthesize the consensus knowledge of an entire community of experts through training on shared datasets. When combined with high-resolution imaging that is becoming more easily acquired from unmanned aerial systems, the collection of this level of image data across many research groups and breeding programs could develop robust training sets for all phenotypes of interest along with the built-in feature of consensus from many experts.

POTENTIAL IMPLICATIONS

The first generation of high-throughput plant phenotyping has focused on sensor and image features that can be directly mapped to plant phenotypes, but remains limited on exploring the full scope of phenotypic variants. Conceptually, deep learning approaches for the next-generation of high-throughput phenotyping can be conceptually extended to any trait of interest in any species for which high-resolution imaging and expert scoring of the phenotypes

can be obtained. This development in high-throughput plant phenotyping can enable breeders and geneticists to measure complex phenotypes on the size of populations that are used in breeding programs and are needed to understand gene function on a genome-wide scale and uncover genetic variants to develop vastly improved varieties for a future of food security.

Figure 1. Phenotyping platform and "breeder-trained" image data sets in this study. (**a**) Aerial view of field-based high-throughput phenotyping platform deployed in current study traversing wheat plots with superimposed example representation of imaging positions and example field plot boundaries. (**b**) imaging array of multiple DSLR cameras deployed on the phenotyping platform to collect geo-referenced proximal images of the wheat canopy. (**c**) combination of images assigned to respective field plots and merged with visual breeder scored to develop the labeled imaged dataset for training the neural networks.

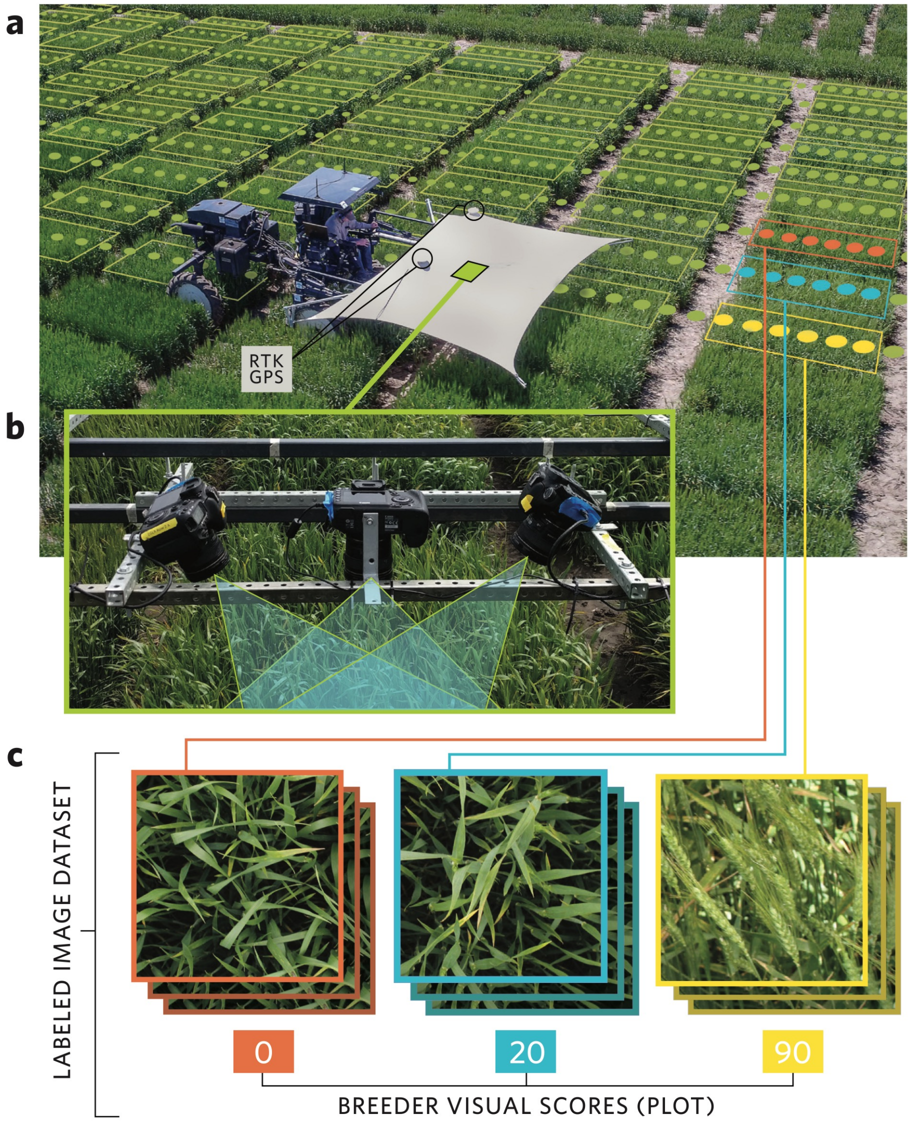

Figure 2. Schematic of the "WheatNet" neural network developed for classifying cropped image patches followed by census voting network for the whole image.

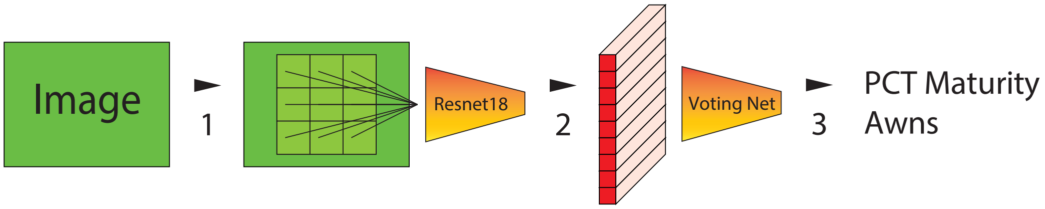

Figure 3. Example of logistic regression to score heading date from time series measurements for a single field plot (17ASH20014). Shown are visual scores (circles) and predictions from the convolutional neural network (squares) and the respective fitted regression lines. The 50% interaction of the regression was identified and scored as the heading date.

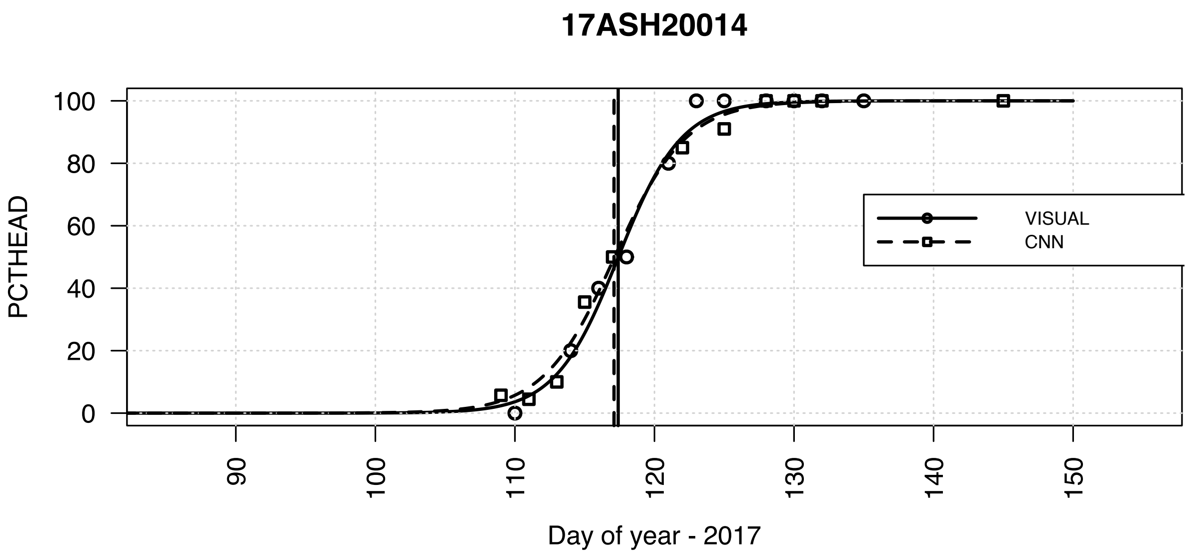

Figure 4. Heading date compared between visual scoring and convolutional neural network (CNN) from logistic regression applied individually to 676 field plots in ‘Lakin x Fuller’ recombinant inbred line population in 2017 for percentage heading from time-series observations (visual scoring) and time-series imaging (CNN predictions). The dates of visual scoring (VISUAL) and imaging data collection (CNN) are shown on the axes.

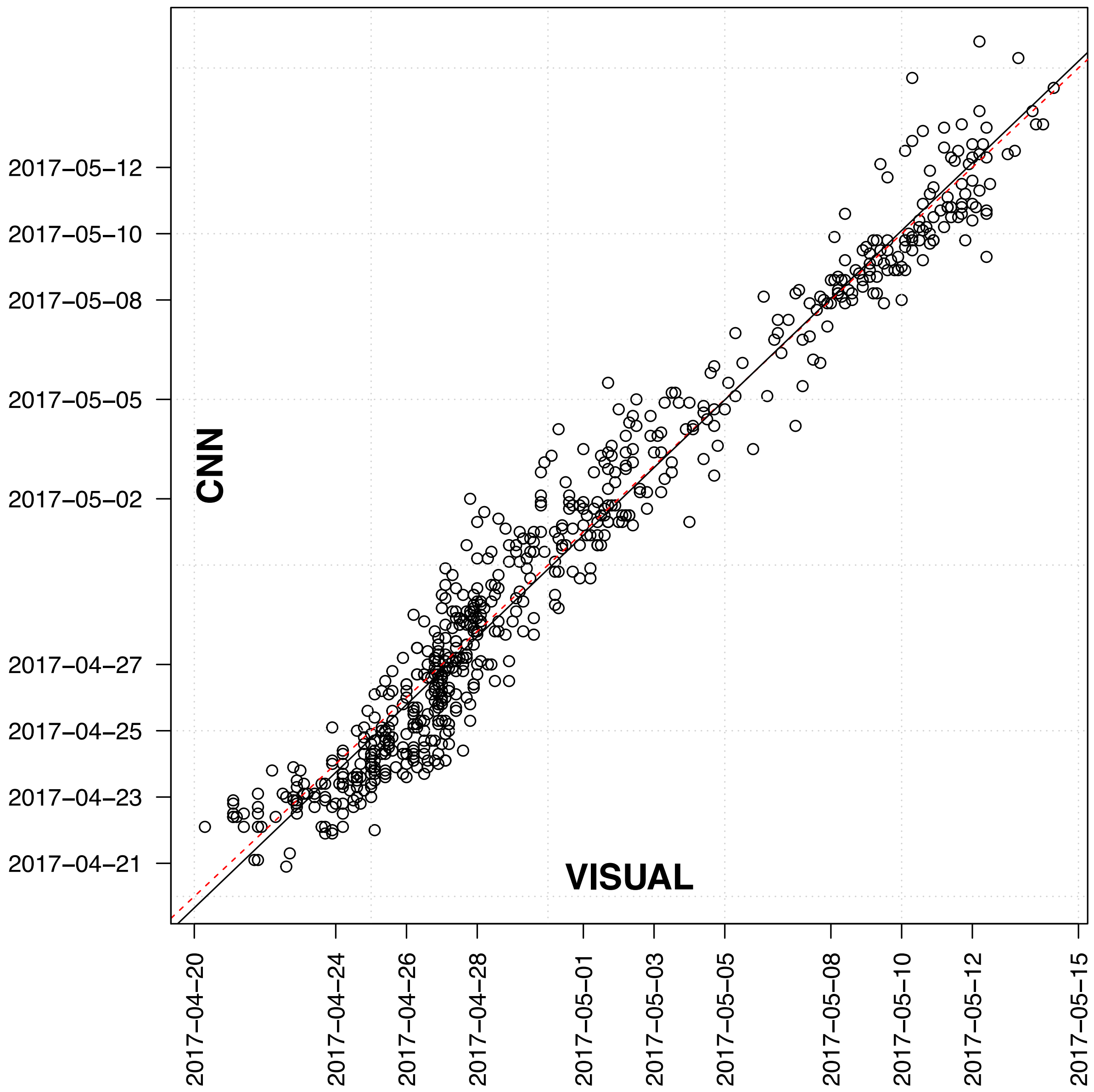

Figure 5. Genetic analysis of heading date scored by deep learning. Genome wide association testing for heading date measured by deep-learning on the Lakin x Fuller recombinant inbred line testing for 21 wheat chromosomes. Markers above Bonferroni multiple-test correction threshold shown in red. Epistatic interactions (internal connections) for two-gene interaction model. Significant interactions at Bonferroni correction show with heat-colors indicating significance level (LOD range from 4.97 (blue) to 22.4 (red)).

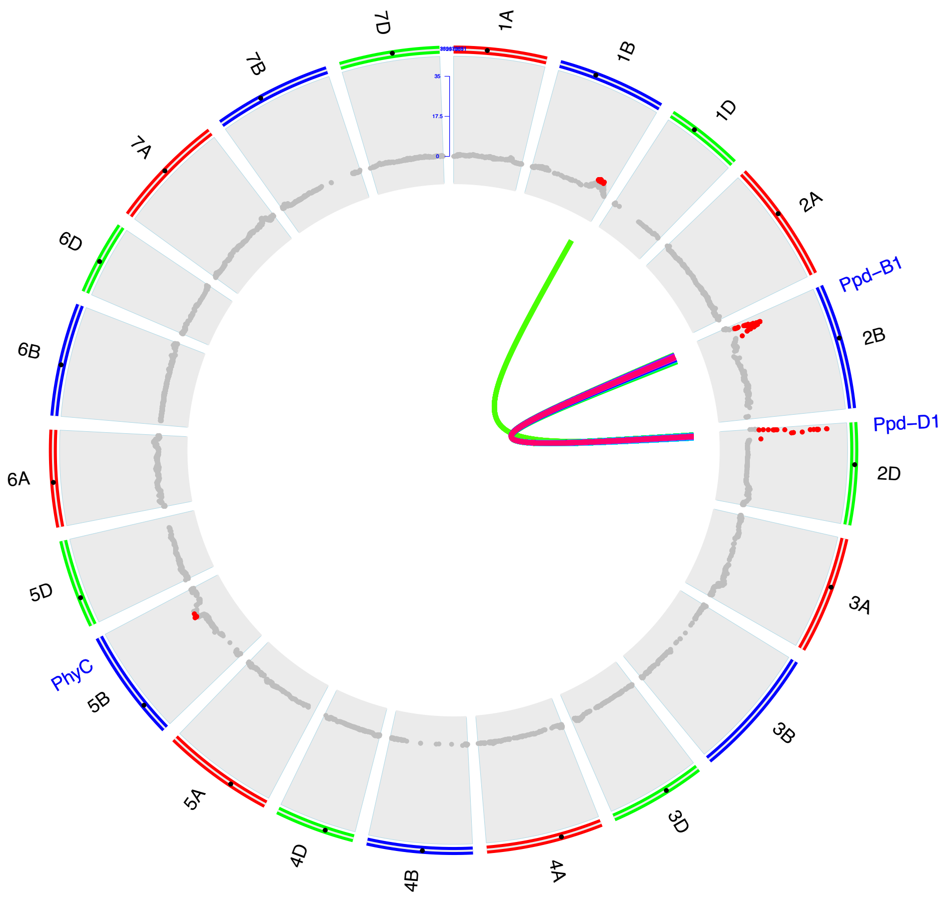

Figure 6. Epistatic interaction of loci controlling heading date. Phenotypic distributions of genotypes at loci showing significant epistatic interactions for heading date measured by CNN predictions including Ppd-D1, Ppd-B1 and QTL identified on Chr. 1B in the Lakin x Fuller RIL population. Plus (+) and minus (-) indicates genotypes with or without the early allele.

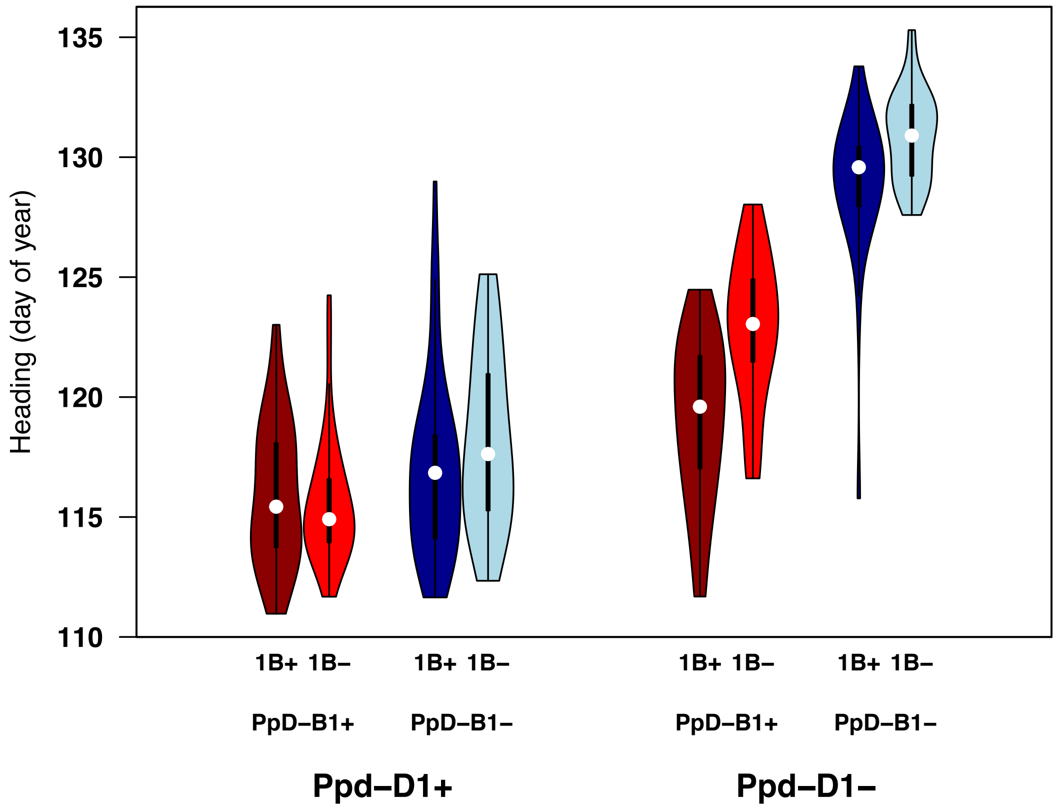

METHODS

Development of field-based high-throughput platform for image collection

A phenotyping mobile unit ("PheMU") [15] was developed to image winter wheat field plots at Kansas State University (KSU), Manhattan, Kansas. PheMU was constructed on a high-clearance sprayer (Bowman Mudmaster, Bowman Manufacturing Co., Inc., Newport, Arkansas, USA) with a height-adjustable sensor boom to capture images throughout the growing season from consistent distance from the canopy. An imaging array including multiple digital single-lens reflex (DSLR) cameras (EOS 7D, Canon) was carried by the PheMU to capture high-resolution crop images. For georeferencing images, two GNSS antennae (AG25, Trimble; Sunnyvale, California, USA) were installed at each end of the sensor boom and connected to an RTK GNSS receiver (BX982, Trimble; Sunnyvale, California, USA). A laptop computer was used to control cameras, collect images, and log positioning data. To reduce the shadows on the canopy and capture images in a balanced light condition, a rectangle-shape shade sail (Kookaburra OL0131REC, Awnings-USA, Camanche, Iowa, USA) was mounted over the sensor boom.

Plant Materials and Field Experiments

Two populations were used in this study, 1) a recombinant inbred line (RIL) population consisting of 318 RILs developed from single seed descent to the F_5 generation from a cross between U.S. winter wheat varieties 'Lakin' and 'Fuller', with seed for field trials increased from a single plant in the F_5 generation, and 2) a diverse panel of winter wheat inbred lines (Diversity Panel) that has been previously described consisting of 299 winter wheat lines of historical and current cultivars [20]. In the present study, we augmented these lines with an additional 41 newly released cultivars (**Supplementary Data S1**).

Field trials were planted at the Kansas State University Ashland Bottoms research farm (39.131595, -96.619524) on 2015-10-10 and 2016-10-18 for the Lakin x Fuller and 2015-10-20 and 2016-10-19 for the diversity panel for the two years, respectively. Trials were planted in two replications of an augmented incomplete design with one check plot per block of 'Lakin'

and 'Fuller' for the Lakin x Fuller population and 'Everest' for the AM Panel (**Supplementary Data S1**)

Phenotypic measurements for awns and percent heading were visually scored and recorded using FieldBook [21]. Awn morphology was scored on the diversity panel according to crop ontology CO_321:0000027 (www.cropontology.org). The Lakin x Fuller population is completely awned. Percent heading was visually scored at two to four day intervals through the season scored corresponding to development stages of Zadoks [16] 49 to 59 (Crop Ontology CO_321:0000476). Percent heading was scored as the percentage of heads emerged from the boot to give a direct indicator with multiple linear classes from 0% to 100% of heading progression to model with the deep learning. A score of 50% heading was given consistent with the standard visual observation of heading date when 50% of the spike is emerged on 50% of all stems (Crop Ontology CO_321:0000840). For imaging timepoints that corresponded to visual measurements on the same date, we assigned labels to all images from a given plot on that date with the visual score for that respective plot.
For imaging timepoints that did not correspond with visual measurement dates, the successive visual measurements for dates directly before and after the imaging date were used to impute the percentage heading labels for images on that date. A weighted average of the visual scores based on number of days from the imaging date was used as follows:

$$PCT_t = (PCT_{t-1} * (T_t - T_{t-1}) + PCT_{t+1} * (T_{t+1} - T_t)) * \frac{1}{(T_{t-1} - T_{t+1})}$$

where *PCT* is the visual score of percentage heading at the corresponding timepoint, *t* is the respective timepoint in days. The timepoints *t-1* and *t+1* correspond to the timepoints of the respective visual scores directly before and after the date of imaging. This gives a weighted average of the two successive visual measurements for imaging timepoints that did not correspond to the same day a visual measurements.

Field Mapping and Image Collection

Proximal imaging of the field plots was also conducted at target intervals of two to three days. Due to rain and wet field conditions, there was a gap in imaging date in 2017 of five days. Cameras in the imaging array were set to capture proximal images of wheat plots in nadir and off-nadir view angles, and from different parts of each plot. The PheMU was operated at 0.3 to 0.5 m/s with the cameras positioned at 0.5m above the canopy. Each DSLR camera was triggered to take mid-size JPEG images (8 megapixels) in 1.25Hz by a C# program using the Canon EOS Digital SDK (EDSDK v2.14, Canon, JPN). (**github.com/xwangksu/CamControl**). To capture unblurry and focused images on a mobile platform, the camera was set using manual focus, 1/500-second shutter speed, and F-5 aperture. The camera ISO was adjustable according to the light condition at the beginning of each image acquisition. Each image was directly transferred from the camera to the laptop computer. Image file names and the time stamps when captured were logged in a text file for subsequent georeferencing. Images were then georeferenced and positioned to individual field plots using the approach of Wang *et al*. [22]. The boundary coordinates of each field plot were delineated in Quantum GIS (QGIS, www.qgis.org) using an orthomosaic field map generated from aerial images using the approach of Haghihatilab *et. al.* 2016 [5]. Images inside each plot boundary were geotagged with designated plot IDs to be linked with the plot levels scores (as described above) and genotype information based on the entry for that respective field plot (**Supplementary Dataset**).

Neural Networks

We followed the standard approach of starting with an existing network pre-trained on the Imagenet dataset [23] and fine-tuning that network to give optimal performance for our task. We subsequently made slight modifications to standard CNN to optimize the network for the phenotyping task. For the baseline model we used Resnet [24], which has been previously used for many applications including people re-identification [25] and flower species identification [26]. Code use for neural networks is available at **https://github.com/littleredxh/WheatNet**.

Training data preparation and size:

For the awned phenotype, training and validation images were from 2017 association mapping panel (AM Panel) containing 700 plots, of which 29 plots were awnless and the remaining were awned. As a validation dataset, we reserved 70 plots from the awned and 5 plots from the awnless and sampled images from the remaining plots as the training data. For the training set, we used 2000 images for awned plots and 1800 images for awnless plots. The "WheatNet" network was trained with the following parameters; mini-batch stochastic gradient with a batch size of 44. The learning rate is initialized at 0.01 and was reduced by 80% every 5 training epochs. Training continued for 30 epochs.

For estimating heading percentage the training dataset is from the AM-Panel image set from 2016 and 2017 which consists 711 plots. In these plots, the training set contains 611 randomly sampled plots, and the validation set contains the remaining 100 plots. Each plot was imaged on multiple dates, and on each date, the multiple images were taken of each plot. Each plot was assigned one of 11 classes, corresponding to a visual score of percentage heading of 0, 10, 20, … 100. To create a training dataset, images from each plot were randomly selected to get 2000 images per class. A validation data set was sampled from images from the 100 plots from the diversity panel by randomly selecting 200 images for each class.

Resnet, and most convolutional neural networks, are limited in the size of the image they can analyze. We cropped the large images captured by the tractor system into 3 by 3 blocks (patches). Each of the 2000 images per class give 9*2000 patches per class. Therefore, overall we trained the networks with 198,000 image patches cropped from 22,000 images. The test dataset comes from the Laken-Fuller RIL population in 2017. This dataset contains about 80,000 images per class, and all images came from 676 plots and germplasm that were never seen in the test or validation data.

To develop a more robust CNN for heading percentage we developed two important modifications of the base network.

Modification 1: Error function that gives partial credit for classifications that are within 10-20% of the label.

The output of a CNN can be viewed as a probability distribution of classes with close percentage classes being more similar. Meanwhile, a given visual labeled maturity percentage can have a +/-10% or +/-20% offset mislabeled condition. In our data, we evaluated repeated visual scoring a small subset of the field plots and estimated the percentage of the mislabeled images which has a discrepancy of 10% at about 10% in each class, and the mislabeled images which has a discrepancy of 20% at about 5% in each class. There was negligible discrepancy of 30% or greater. Therefore, the image label was modeled as a distribution that the labeled class has the value 0.7 for correct class, 0.1 for 10% discrepancy and 0.05 for 20% discrepancy respectively and the remaining classes have value 0, which ensure the sum of all class probability is 1. The error function calculates the average mismatch of the probability of output and the target distribution, which calculate the absolute difference of each class value between output and the target value.

Modification 2: WheatNet

In order to keep as much of the full details of the images as possible, which is sensitive to maturity classification, the modified architecture keeps the input image with the resolution of 672 by 672 pixel. The main idea of the design of the architecture is to mimic how experienced individuals assign a phenotype (e.g. the maturity percentage) from a wheat plot or image, by taking a consensus from viewing all part of the images. To capture this feature of visual scoring in the CNN, the network classified the maturity percentage of each image patch and then summarizing all predictions and giving output for the concensus prediction for a whole image. The validation dataset for each network was used to determine the optimal number of training epochs, hyper-parameters. Full detail of each layer in the Wheat Net is included as **Supplementary Table S3**.

Genetic Analysis

RILs from the Lakin-Fuller population were genotyped using genotyping-by-sequencing with two-enzymes, *Pst*I and *Msp*I [27]. Two sets of libraries for the RILs and replicated samples of the parents were made in 95-plexing and 190-plexing and sequenced with Illumina HiSeq2000 and NextSeq, respectively. Single nucleotide polymorphisms (SNPs) were called using TASSEL 5 GBS v2 pipeline [28] anchored to the IWGSC wheat genome v1.0 assembly (https://wheat-urgi.versailles.inra.fr/Seq-Repository/Assemblies) with the following parameters: -mnQS 10, enzyme PstI-MspI, -c 20, -minMAPQ 20, and -mnMAF 0.1. Unique sequence tags were mapped to the wheat reference genome (Chinese Spring) using bowtie2 [29] with the following settings: --end-to-end -D 20 -R 3 -N 0 -L 10 -i S,1,0.25. SNPs passing at least one criteria were recovered: inbreeding coefficient of at least 0.8, Fisher Exact test (P<0.001) to determine bi-allelic single locus SNPs[27] and Chi-square test for bi-allelic segregation with 96% expected inbreeding. SNPs having two parents homozygous within and polymorphic between were extracted and missing loci were imputed with LB-impute [30] with parameter settings of -readerr 0.1 -genotypeerr 0.1 -window 7. Finally, SNP sites were removed if minor allele frequency (MAF) < 0.1, missing > 30% or heterozygosity > 6%. The TASSEL pipeline gave approximately 82% useable reads out of 2.15 billion reads. The overall alignment of 1,973,081 unique tags against the reference genome was 91% with a unique alignment of 37.8%. A total of 44,679 SNPs was discovered out of which 28,972 passed filtering. We then filtered RILs for missing data and heterozygosity resulting in 306 RILs and the two parents with suitable geneotypes. Finally, 8,797 SNPs were recovered after imputation with the additional filtering for MAF, missing and heterozygosity. All raw sequencing data for the Lakin x Fuller RIL population is available from NCBI SRA under accession number **SRP136362**.

Using traits directly from visual scoring and from the network image classification by the K-net we calculated trait distributions, variance components, and best linear unbiased predictors in R statistical software [31] (Supplemental Information)

To model heading date from time series scoring/predictions of percent heading, we fit a logistic growth curve model for each individual plot according to the function:

$$y_i = \frac{phi1}{1 + e^{-(phi2 + phi3 * day)}}$$

Where y_i is the is the ith observation of heading percentage for a given plot. *phi1* is the asymptote maximum and was set to fixed at 100 for maximum percent heading. This model allows for different rates of development through heading as defined by $phi2$ and $phi3$. The independent variable day was calculated as the day of the year for observation *i*. The model was fit using nls function from nlme package [32]. To increase the robustness of fit we added points of 0 and 100 percent heading at 10, 20 and 30 days before the first and after the last visual measurements, respectively, corresponding to dates when all of the plots where not started and completely headed. The heading date for each plot was calculated as the date closest to 50% using the predict function in R at 0.1 day increments over the full range of the days.

For heading date, phi2 and phi3, we calculated broad-sense heritability on a line mean basis according to Holland *et al.*[33] for replicated trials of inbred lines (e.g. clonal species) in one location within one year as:

$$H^2 = \frac{\sigma_G^2}{\sigma_G^2 + \frac{\sigma_e^2}{r}}$$

where σ_G^2 is the total genetic variance for entries in the trial, $\sigma_e^2$is the error variance and r is the number of replications. For heritability estimation across multiple years at one location as:

$$H^2 = \frac{\sigma_G^2}{\sigma_G^2 + \frac{\sigma_{GY}^2}{y} + \frac{\sigma_e^2}{yr}}$$

where σ_{GY}^2 is the genotype by year variance and y is the number of years evaluated. Variance components were estimated by fitting mixed models in asreml package [34] in R. Models were fit with random effects for entry, year and replication within year and using a row-column autoregressive variance structure using the following model:

$$y_{ijk} = g_i + y_j + r_{k(j)} + e$$

where, y_{ijk} is the observed plot-level phenotype, g_i is the random effect genotype effect of entry i distributed as iid where $g_i \sim N(0, \sigma_i^2)$, y_j is the random effect for year j, $r_{k(j)}$ is the random effect of replication k within year j, and e is the residual variance partitioned with a two-dimensional autoregressive spatial structure (AR1 ⊗ AR1). Best linear unbiased estimates (BLUEs) were estimated for each entry within and across years by fitting the same model with entry as a fixed effect and using the predict function in R.

Following calculation of heading date, we observed a bimodal distribution of heading dates from the multi-year model BLUPs (**Supplemental Figure S4**). The distribution was delimited at 124 days to calculate the number of 'early' and 'late' lines. The number of lines in each group was fit to a χ^2 test for two classes with probably of 0.75 and 0.25 according to a two-gene dominant epistasis model for inbred lines using the chisq.test function in R.

We tested for genetic association of heading date in the Lakin x Fuller population as measured by the logistic regression using a standard mixed model:

$$y = Wv + X\beta + Zu + e$$

where y is the projected phenotype of heading date (50% intersect) or rate of heading (phi3) from the logisitic regression models. The use of a bi-parental population without population structure or kinship greatly simplified the equation to:

$$y_i = \beta_{i,k} + e$$

where $\beta_{i,k}$ is the allele substitution effect for locus k in individual i; and e is residual error. Each marker effect was estimated using the lmer function in R and Bonferroni correction for multiple testing correction of experimental alpha of 0.05.

Following identification of significant marker association, we tested for two-way epistatic interactions for all markers that were associated with heading date using the model:

$$y_i = \alpha_{i,j} + \beta_{i,k} + \varepsilon_{i,j \cdot k} + e$$

where, $y_i$is the phenotype of the individual i, $\alpha_{i,j}$ is the allele substitution effect for locus j in individual i; $\beta_{i,k}$ is the allele substitution effect for locus k in individual i; $\varepsilon_{i,j \cdot k}$is the interaction between locus j and k; and e is residual error.

Code used for genetic analysis is available at **https://github.com/jessepoland/htp-analysis**

ACKNOWLEDGEMENTS

We sincerely appreciate the assistance of Shuangye Wu in genotyping, Mark Lucas in data curation, and Haley Ahlers in graphics design, along with all members of the Wheat Genetics Lab at Kansas State University for project support, input and feedback. This work was supported by the National Science Foundation (NSF) Plant Genome Research Program (PGRP) (Grant No. IOS-1238187), the Kansas Wheat Commission and Kansas Wheat Alliance, the US Agency for International Development (USAID) Feed the Future Innovation Lab for Applied Wheat Genomics (Cooperative Agreement No. AID-OAA-A-13-00051), and by the NIFA International Wheat Yield Partnership (Grant No. 2017-67007-25933/project accession no. 1011391) from the USDA National Institute of Food and Agriculture. The opinions expressed herein are those of the author(s) and do not necessarily reflect the views of the U.S. Agency for International Development, the U.S. National Science Foundation, or the U.S. Department of Agriculture. The funders had no roll in study design, data collection or analysis.

AUTHOR CONTRIBUTIONS

J.P. conceived and designed the study. B.E. managed the field trials and collected phenotypic data. X.W. developed the phenotyping platform and collected all image data. H.W. and R.P. analyzed images and developed neural networks. S.S. analyzed genetic data. J.P. directed the overall project, analyzed genetic and phenotypic data. J.P., X.W., H.W. and R.P. wrote the manuscript. All authors reviewed and approved the manuscript.

COMPETING INTEREST

The authors declare no competing interests.

The plant materials tested in this study are public germplasm and/or were tested in accordance with local, national and international guidelines and legislation with the appropriate permissions and/or licenses for the study the present study.

References:

1. Tester M and Langridge P. Breeding technologies to increase crop production in a changing world. *Science*. 2010;327(5967):818-22.
2. Furbank RT and Tester M. Phenomics – technologies to relieve the phenotyping bottleneck. *TIPS*. 2011;16(12):635-44.
3. Andrade-Sanchez P, Gore MA, Heun JT, et al. Development and evaluation of a field-based high-throughput phenotyping platform. *Functional Plant Biology*. 2013;41(1):68-79.
4. Pauli D, Andrade-Sanchez P, Carmo-Silva AE, et al. Field-based high-throughput plant phenotyping reveals the temporal patterns of quantitative trait loci associated with stress-responsive traits in cotton. *G3*. 2016;
5. Haghighattalab A, González Pérez L, Mondal S, et al. Application of unmanned aerial systems for high throughput phenotyping of large wheat breeding nurseries. *Plant Methods*. 2016;12(1):1-15.
6. LeCun Y, Bengio Y and Hinton G. Deep learning. *nature*. 2015;521(7553):436.
7. Krizhevsky A, Sutskever I and Hinton GE. Imagenet classification with deep convolutional neural networks. In: *Advances in neural information processing systems* 2012, pp.1097-105.
8. Ubbens JR and Stavness I. Deep plant phenomics: A deep learning platform for complex plant phenotyping tasks. *Frontiers in plant science*. 2017;8(1190.
9. Pound MP, Atkinson JA, Townsend AJ, et al. Deep machine learning provides state-of-the-art performance in image-based plant phenotyping. *Gigascience*. 2017;6(10):1-10.
10. Dobrescu A, Valerio Giuffrida M and Tsaftaris SA. Leveraging multiple datasets for deep leaf counting. In: *Proceedings of the IEEE International Conference on Computer Vision* 2017, pp.2072-9.
11. Giuffrida MV, Doerner P and Tsaftaris SA. Pheno-deep counter: A unified and versatile deep learning architecture for leaf counting. *The Plant Journal*. 2018;96(4):880-90.
12. Ghosal S, Blystone D, Singh AK, et al. An explainable deep machine vision framework for plant stress phenotyping. *PNAS*. 2018;115(18):4613.
13. Uzal LC, Grinblat GL, Namías R, et al. Seed-per-pod estimation for plant breeding using deep learning. *Computers and Electronics in Agriculture*. 2018;150(196-204.
14. Hasan MM, Chopin JP, Laga H, et al. Detection and analysis of wheat spikes using convolutional neural networks. *Plant Methods*. 2018;14(1):100.
15. Barker J, Zhang N, Sharon J, et al. Development of a field-based high-throughput mobile phenotyping platform. *Computers and Electronics in Agriculture*. 2016;122(74-85.
16. Zadoks JC, Chang TT and Konzak CF. A decimal code for the growth stages of cereals. *Weed research*. 1974;14(6):415-21.
17. DeChant C, Wiesner-Hanks T, Chen S, et al. Automated identification of northern leaf blight-infected maize plants from field imagery using deep learning. *Phytopathology*. 2017;107(11):1426-32.
18. Poland J and Nelson R. In the eye of the beholder: The effect of rater variability and different rating scales on qtl mapping. *Phytopathology*. 2011;101(2):290-8.

19. Han SS, Park GH, Lim W, et al. Deep neural networks show an equivalent and often superior performance to dermatologists in onychomycosis diagnosis: Automatic construction of onychomycosis datasets by region-based convolutional deep neural network. *PLoS ONE*. 2018;13(1):e0191493.
20. Grogan SM, Anderson J, Baenziger PS, et al. Phenotypic plasticity of winter wheat heading date and grain yield across the us great plains. *Crop Sci*. 2016;56(5):2223-36.
21. Rife TW and Poland JA. Field book: An open-source application for field data collection on android. *Crop Sci*. 2014;54(4):1624-7.
22. Wang X, R. Thorp K, W. White J, et al. Approaches for geospatial processing of field-based high-throughput plant phenomics data from ground vehicle platforms. *Transactions of the ASABE*. 2016;59(5):1053.
23. Deng J, Dong W, Socher R, et al. Imagenet: A large-scale hierarchical image database. In: *Computer Vision and Pattern Recognition, 2009 CVPR 2009 IEEE Conference on* 2009, pp.248-55. IEEE.
24. He K, Zhang X, Ren S, et al. Deep residual learning for image recognition. In: *Proceedings of the IEEE conference on computer vision and pattern recognition* 2016, pp.770-8.
25. Hermans A, Beyer L and Leibe B. In defense of the triplet loss for person re-identification. *arXiv preprint arXiv:170307737*. 2017;
26. Nguyen TTN, Van Tuan Le TLL, Vu H, et al. Flower species identification using deep convolutional neural networks. *AUN/SEED-Net Regional Conference for Computer and Information Engineering*. 2016.
27. Poland JA, Brown PJ, Sorrells ME, et al. Development of high-density genetic maps for barley and wheat using a novel two-enzyme genotyping-by-sequencing approach. *PLoS ONE*. 2012;7(2):e32253.
28. Glaubitz JC, Casstevens TM, Lu F, et al. Tassel-gbs: A high capacity genotyping by sequencing analysis pipeline. *PLoS ONE*. 2014;9(2):e90346.
29. Langmead B, Trapnell C, Pop M, et al. Ultrafast and memory-efficient alignment of short DNA sequences to the human genome. *Genome Bio*. 2009;10(3):1-10.
30. Fragoso CA, Heffelfinger C, Zhao H, et al. Imputing genotypes in biallelic populations from low-coverage sequence data. *Genetics*. 2016;202(2):487.
31. R Core Team. R: A language and environment for statistical computing. Vienna, Austria: R Foundation for Statistical Computing, 2016.
32. Pinheiro J, Bates D, DebRoy S, et al. Nlme: Linear and nonlinear mixed effects models. R package version 3.1-131 ed. 2017.
33. Holland J, Nyquist W and Cervantes-Martinez C. Estimating and interpreting heritability for plant breeding: An update. *Plant Breed Rev*. 2003;22(
34. Butler D. Asreml: Asreml() fits the linear mixed model. R package version 3.0. ed. 2009.

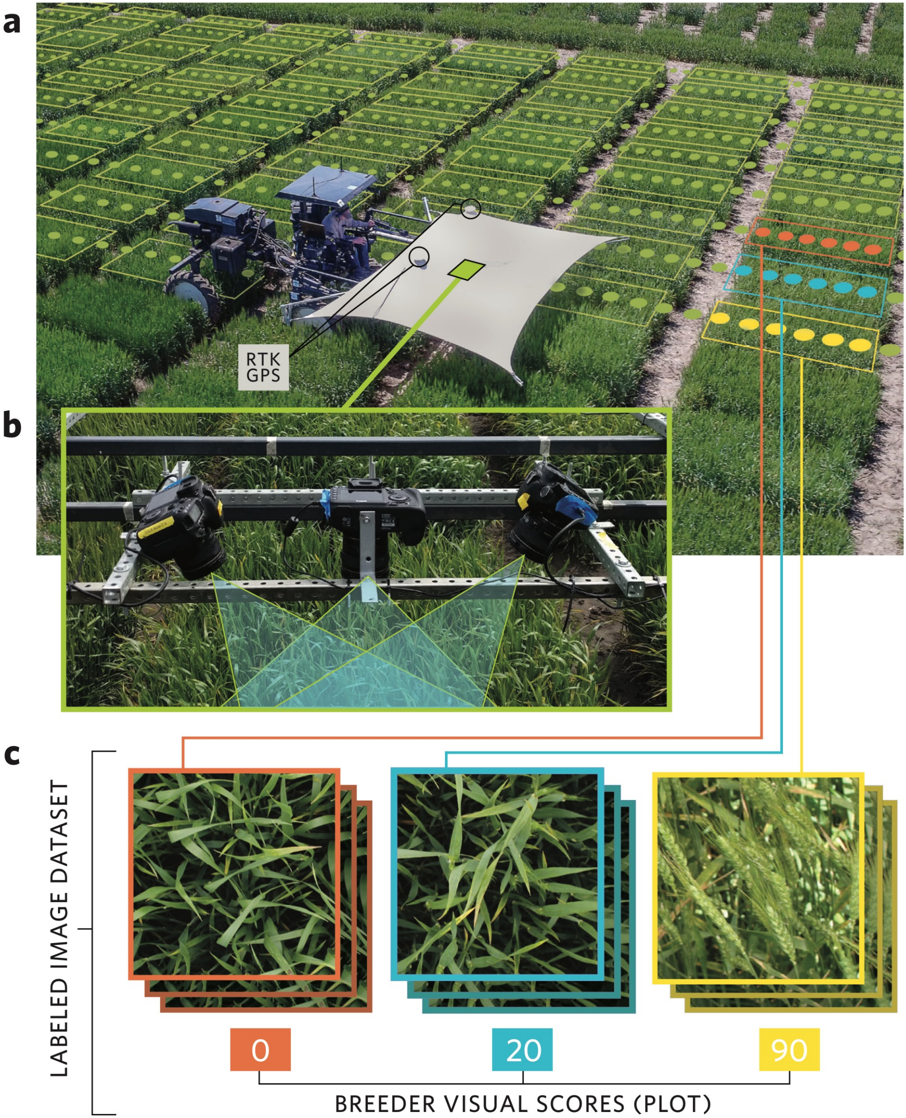

a
RTK
GPS
b
c
LABELED IMAGE DATASET
0
20
90
BREEDER VISUAL SCORES (PLOT)

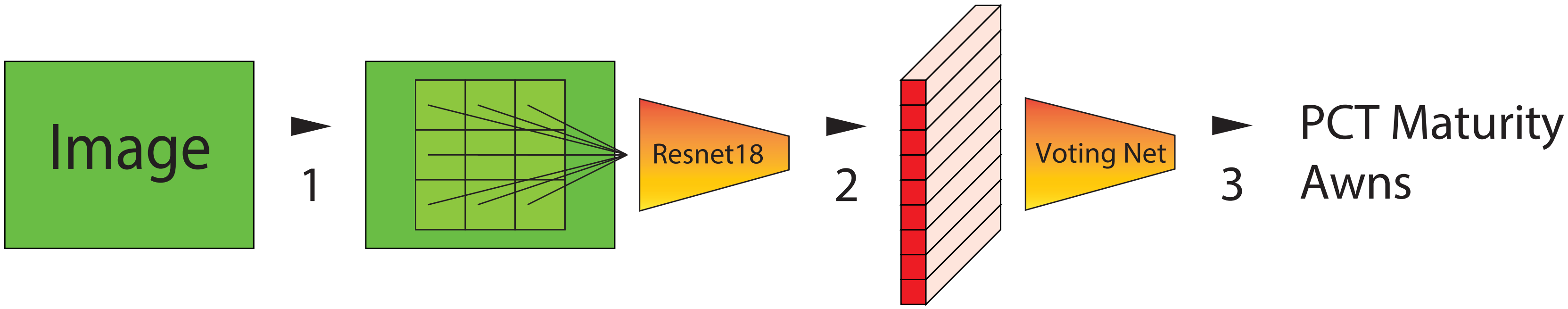
Image
1
Resnet18
2
Voting Net
3
PCT Maturity
Awns

Figure 3

Click here to access/download;Figure;Figure-3_LogReg.pdf

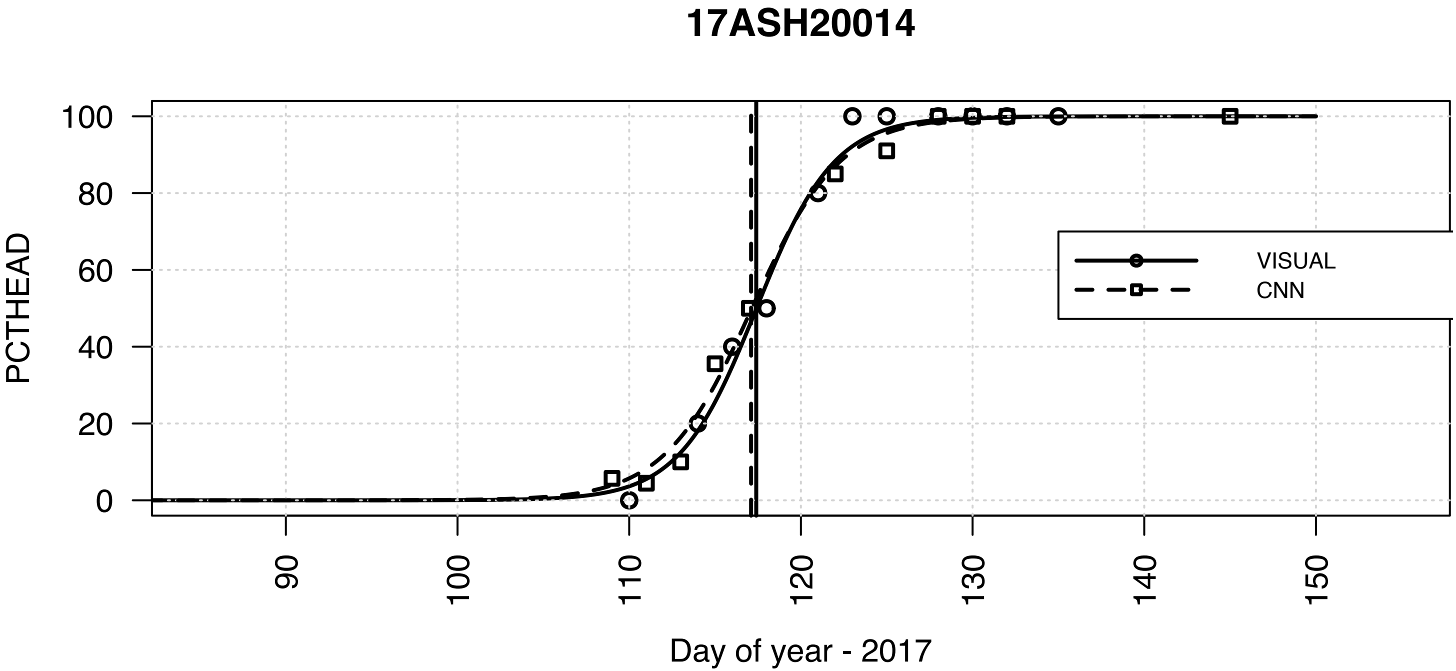

Figure 4

Click here to access/download;Figure;Figure-4_VISvCNN.pdf

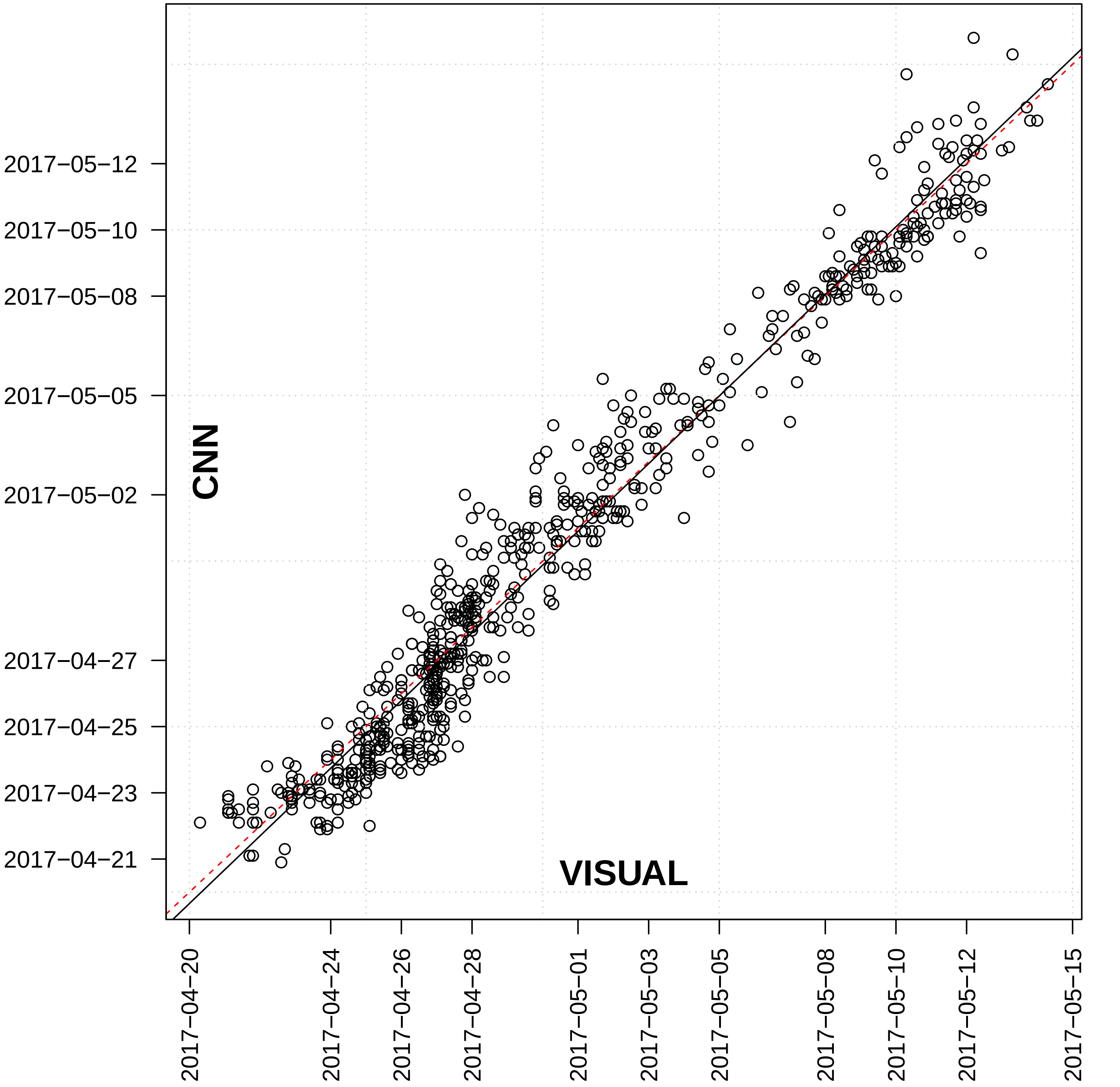

Figure 5

Click here to access/download;Figure;Figure-

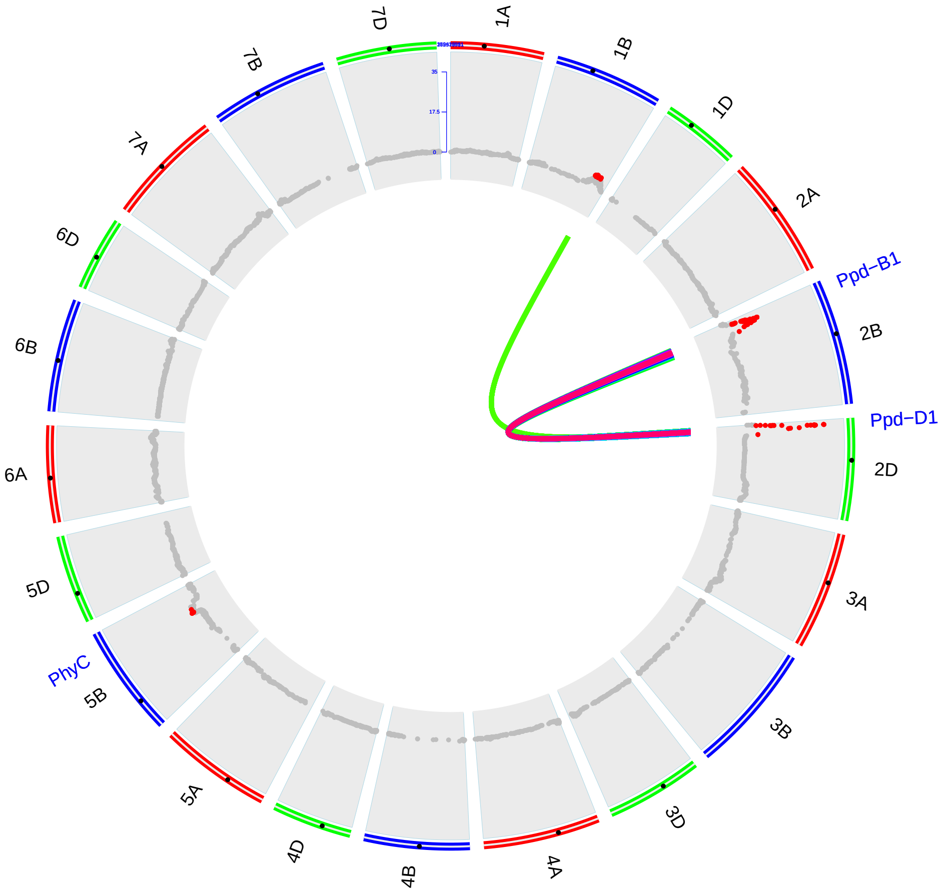

Figure 6

Click here to access/download;Figure;Figure-6_InteractionPlot.pdf

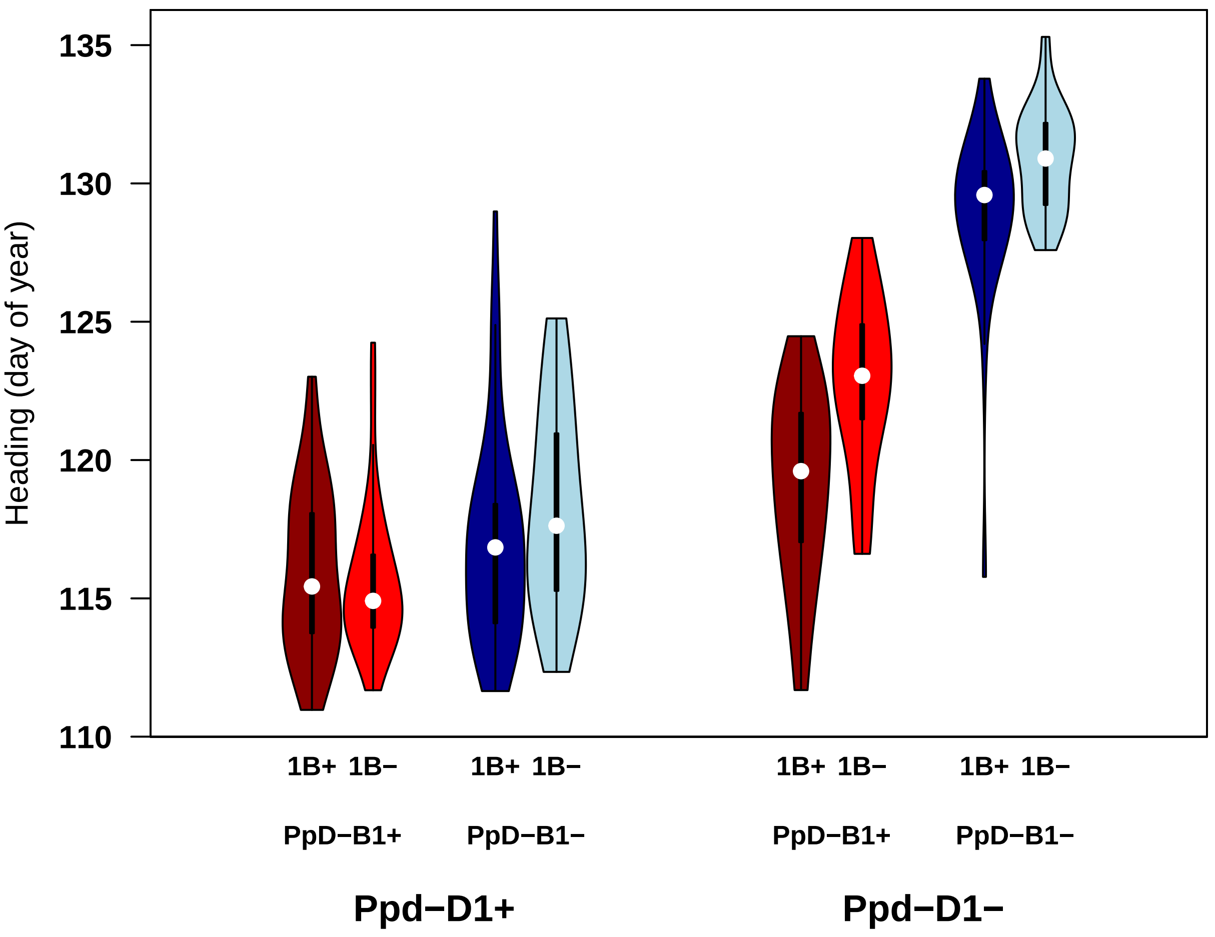

Click here to access/download
Supplementary Material
Supplemental-Document_DeepWheat_20190524.pdf

manuscript with tracked changes

Click here to access/download
Supplementary Material
Manuscript-GigaScience_DeepWheat_REVISION-20190524.docx

review response

Click here to access/download
Supplementary Material
REVIEW-RESPONSE_GigaScience_20190524.pdf
