# Supplementary material for: High-throughput phenotyping with deep learning gives insight into the genetic architecture of flowering time in wheat
Source: Gigascience. 2019 Nov 19;8(11):giz120. doi: 10.1093/gigascience/giz120 (PMC6862935; doi:10.1093/gigascience/giz120)
Supplement: giz120_Supplemental_Files [file giz120_supplemental_files.zip › Supplemental-Document_DeepWheat_20190524.pdf]

SUPPLEMENTAL FIGURES

Figure S1. Example of geolocations of images from one camera (yellow points) marked on the field map (white polygons on the orthomosaic photo). GIS datasets (shape files) for all positioned images are included as supplemental data.

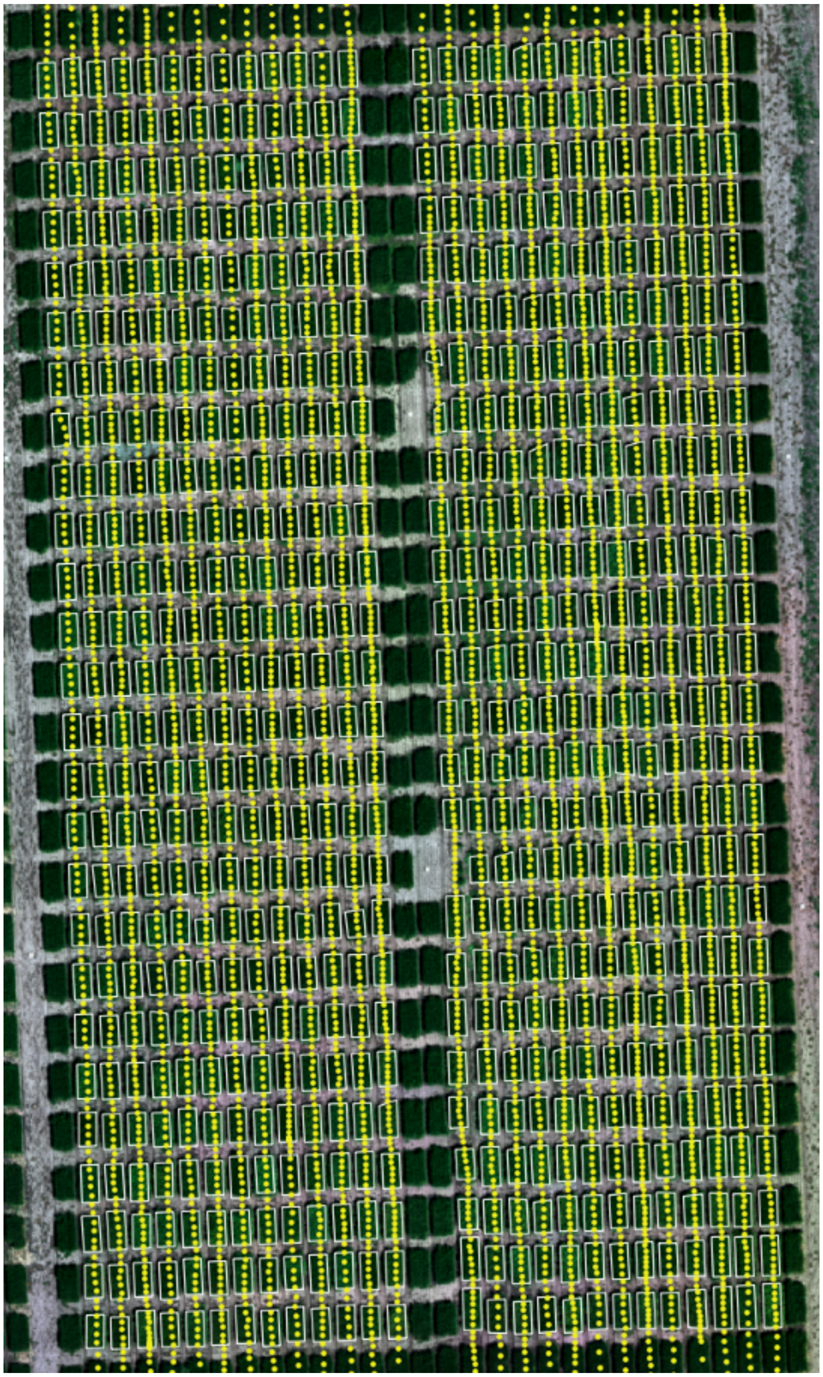

Figure S2. Example of (a) awnless and (b) awned phenotypes from the image dataset for the diversity panel. (c & d) Image examples of misclassified entry 'MFA-2018' showing an heterogenous atypical awnlette phenotype

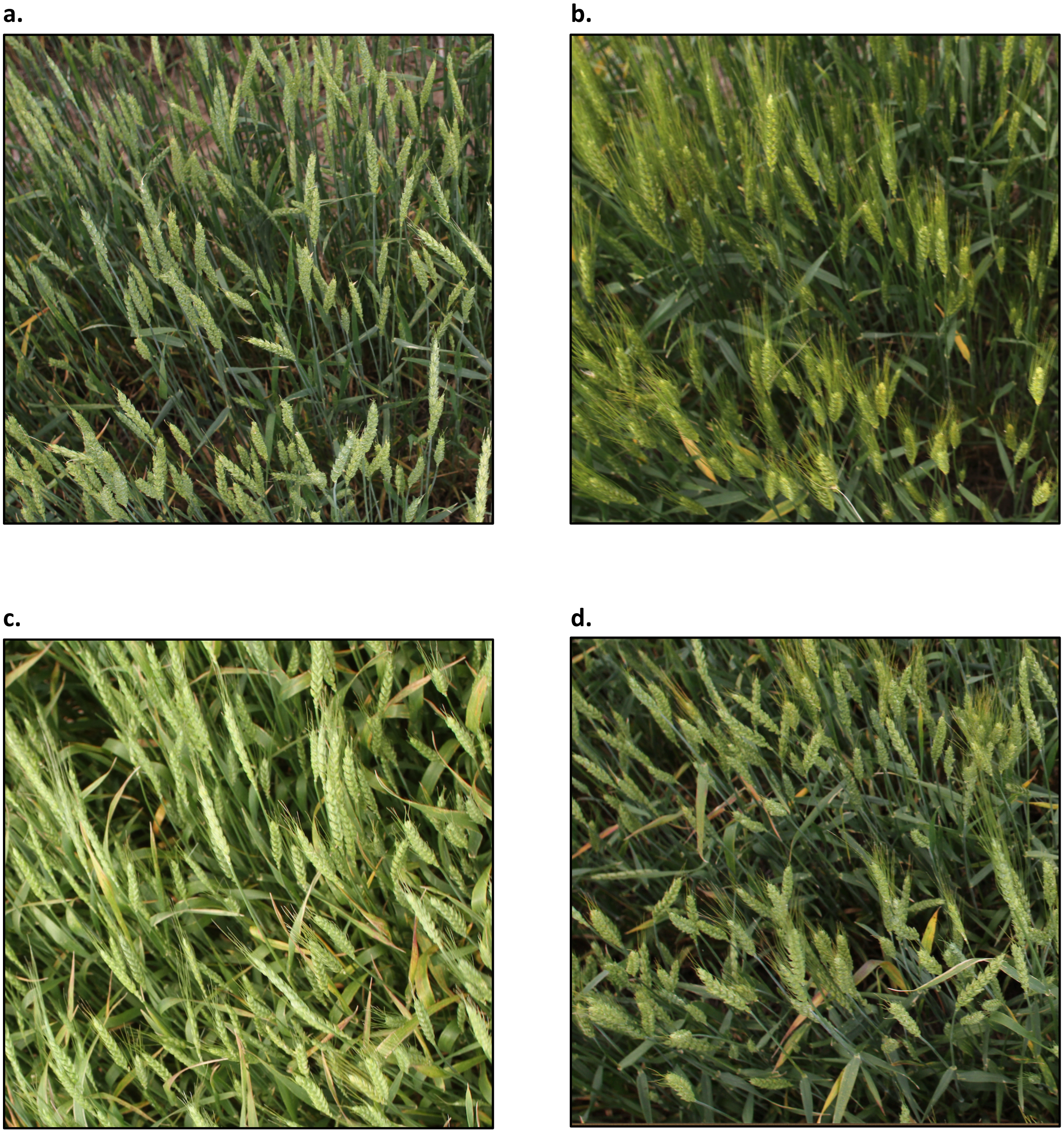

Figure S3. The confusion matrix for (a) training, (b) validation, and (c) testing the CNN for predicting percentage heading.

a.

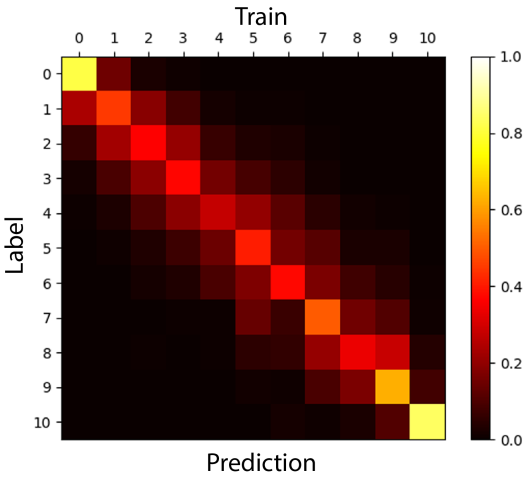

b.

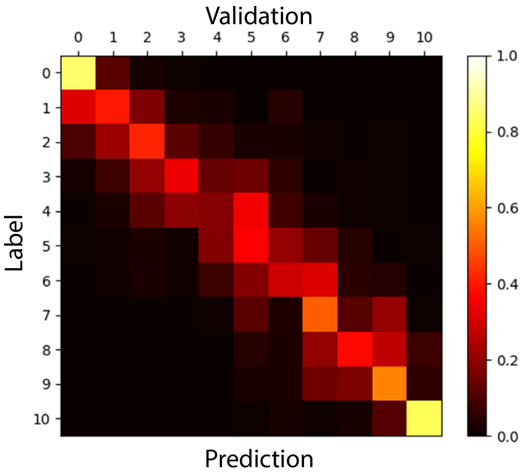

c.

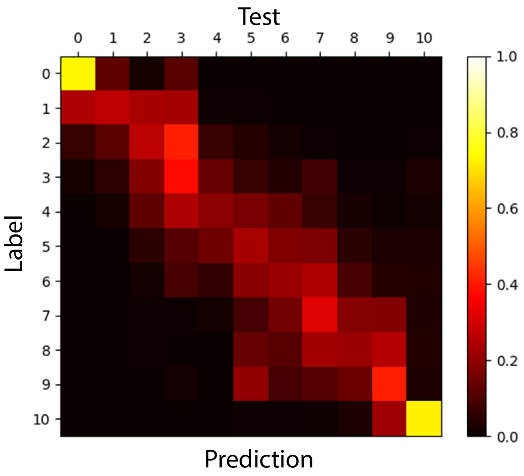

Figure S4. Phenotypic distribution of Lakin x Fuller recombinant inbred lines for heading date (day of year) in 2017 field trial.

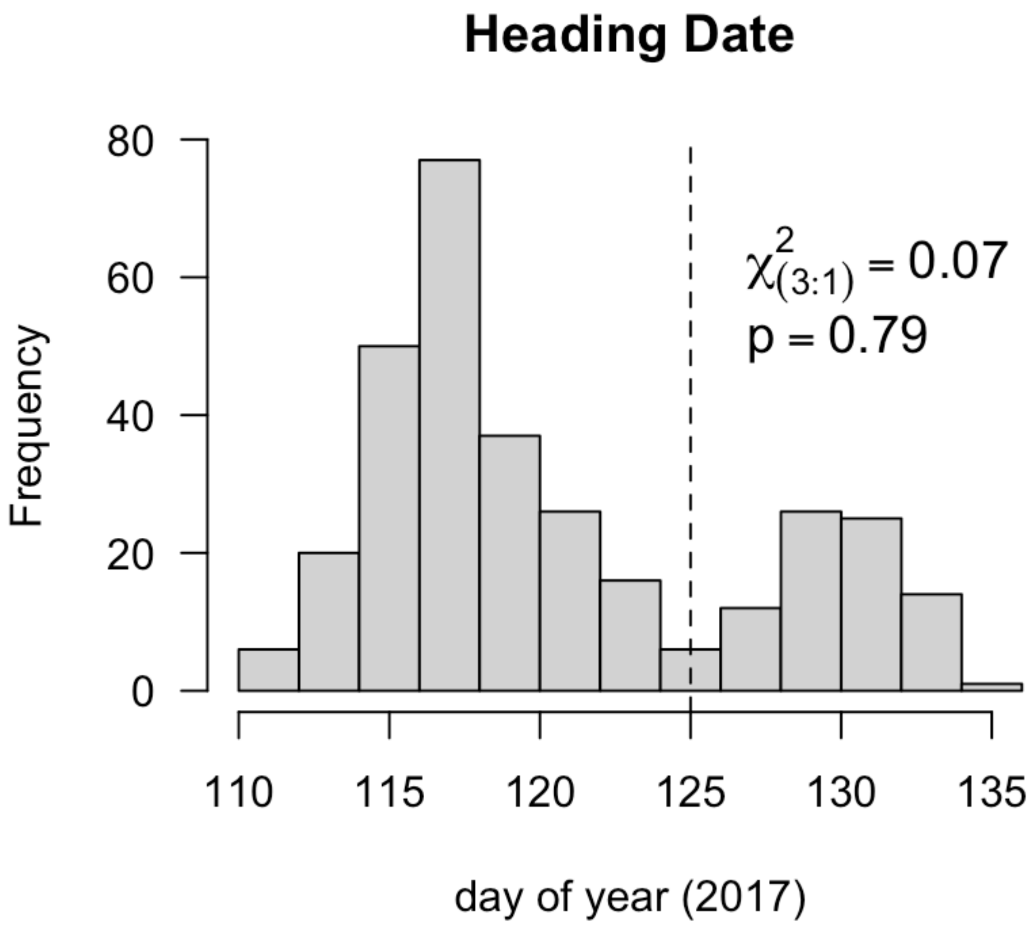

Figure S5. Genome wide association testing of phi2 (slope of logistic regression). No significant markers above Bonferroni multiple-test correction threshold.

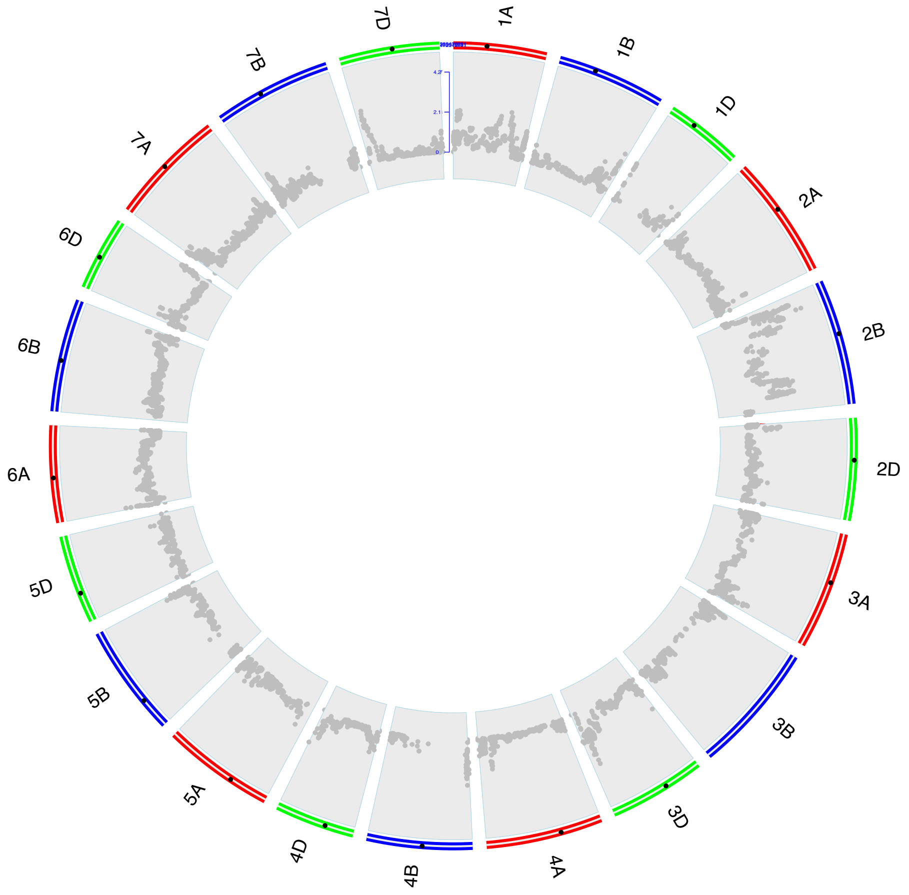

SUPPLEMENTAL TABLES

Table S1. Individual image predictions (a) and accuracy (b) for awns phenotypes

a)

		Predicted	
		AWNED	AWNLESS
**Visual**	AWNED	10944	122
	AWNLESS	19	1419

b)

		Predicted	
		AWNED	AWNLESS
**Visual**	AWNED	0.988	0.011
	AWNLESS	0.013	0.986

Table S2. Supplemental dataset table of field plot entries and field design for Diversity Panel and Lakin x Fuller Recombinant Inbred Line (RIL) populations in 2016 and 2017.

Supplemental Table S3. Full details of each layer in the WheatNet.

Net	Block	Layer	function	in	out	parameters	Layer	function	in	out	parameters
ResNet		layer1	conv_7x7	9xNx3x224x224	9xNx64x112x112	9408					
			bn_2D	9xNx64x112x112	9xNx64x112x112	128					
			relu	9xNx64x112x112	9xNx64x112x112						
			maxpool_3x3	9xNx64x112x112	9xNx64x56x56						
	Block_1	layer2	conv_3x3	9xNx64x56x56	9xNx64x56x56	73728	downsampler	conv_1x1	9xNx64x56x56	9xNx64x56x56	4096
			bn_2D	9xNx64x56x56	9xNx64x56x56	128					
			relu	9xNx64x56x56	9xNx64x56x56						
			drop_out					bn_2D	9xNx64x56x56	9xNx64x56x56	128
		layer3	conv_3x3	9xNx64x56x56	9xNx64x56x56	36864					
			bn_2D	9xNx64x56x56	9xNx64x56x56	128					
			drop_out								
	Block_2	layer4	conv_3x3	9xNx64x56x56	9xNx128x28x28	73728	downsampler	conv_1x1	9xNx64x56x56	9xNx128x28x28	8192
			bn_2D	9xNx128x28x28	9xNx128x28x28	256					
			relu	9xNx128x28x28	9xNx128x28x28						
			drop_out					bn_2D	9xNx128x28x28	9xNx128x28x28	256
		layer5	conv_3x3	9xNx128x28x28	9xNx128x28x28	147456					
			bn_2D	9xNx128x28x28	9xNx128x28x28	256					
			drop_out								
	Block_3	layer6	conv_3x3	9xNx128x28x28	9xNx256x14x14	294912	downsampler	conv_1x1	9xNx128x28x28	9xNx256x14x14	32768
			bn_2D	9xNx256x14x14	9xNx256x14x14	512					
			relu	9xNx256x14x14	9xNx256x14x14						
			drop_out					bn_2D	9xNx256x14x14	9xNx256x14x14	512
		layer7	conv_3x3	9xNx256x14x14	9xNx256x14x14	589824					
			bn_2D	9xNx256x14x14	9xNx256x14x14	512					
Summarization		layer8	avgpooling_7x7	9xNx256x14x14	9xNx256x1x1						
		layer9	fc	Nx9x256	Nx9x11	2816					
		layer10	avgpooling_9x1	Nx9x11	Nx1x11						
		layer11	fc	Nx11	Nx11	121					
						1276729					

Table S4. Analysis of variance for full interaction model of Ppd-D1, Ppd-B1 and locus on 1B.

test = lm(Y~g.2B*g.2D*g.1B)

Analysis of Variance

	Df	Sum Sq	Mean Sq	F value	Pr(>F)
**g.2B**	1	2486	2486	259.7034	< 2.2e-16 ***
**g.2D**	1	5587.1	5587.1	583.6726	< 2.2e-16 ***
**g.1B**	1	241.7	241.7	25.2465	8.887e-07 ***
**g.2B:g.2D**	1	1026.8	1026.8	107.2628	< 2.2e-16 ***
**g.2B:g.1B**	1	1.5	1.5	0.155	0.694053
**g.2D:g.1B**	1	99.3	99.3	10.3704	0.001428 **
**g.2B:g.2D:g.1B**	1	48.7	48.7	5.0921	0.024787 *
**Residuals**	287	2747.3	9.6		

Signif. codes: 0 ‘***’ 0.001 ‘**’ 0.01 ‘*’ 0.05 ‘.’ 0.1 ‘ ’ 1
