# Supplementary material for: High-throughput phenotyping with deep learning gives insight into the genetic architecture of flowering time in wheat
Source: Gigascience. 2019 Nov 19;8(11):giz120. doi: 10.1093/gigascience/giz120 (PMC6862935; doi:10.1093/gigascience/giz120)
Supplement: giz120_Response_to_Reviewer_Comments_Revision_1 [file giz120_response_to_reviewer_comments_revision_1.pdf]

Author's Response To Reviewer Comments

Close

attached supplemental document

Close
