# Supplementary material for: High-throughput phenotyping with deep learning gives insight into the genetic architecture of flowering time in wheat
Source: Gigascience. 2019 Nov 19;8(11):giz120. doi: 10.1093/gigascience/giz120 (PMC6862935; doi:10.1093/gigascience/giz120)
Supplement: giz120_Reviewer_1_Report_Original_Submission — Valerio Giuffrida -- 2/7/2019 Reviewed [file giz120_reviewer_1_report_original_submission.pdf]

Reviewer Report

Title: High-throughput phenotyping with deep learning gives insight into the genetic architecture of flowering time in wheat

Version: Original Submission **Date:** 2/7/2019

Reviewer name: Valerio Giuffrida

Reviewer Comments to Author:

In this paper, the authors use a deep neural network to predict awn phenotype and percentage heading in the wheat. Although the paper is written in a good English, the overall organisation and methodology is rather hard to follow.

From the CNN perspective, it is unclear what the input is (a cropped image for my understanding), and it is even more unclear what the outputs are. In one case, they train to predict the awn phenotype, but what does it mean? Is the network trained to find awned and awnless genotype (binary classification?). When they predict the percentage heading, is this another network? Moreover, why they use classification, instead of regression, to predict the percentage heading? I believe continuous number prediction might fit better in this context.

As a follow up, in line 89, what are these breeders score the authors refers to?

By looking at the results of awn and awnless plants, an accuracy of the ~98% indicates that the problem is rather easy for the DNN. My experience in computer vision, in particular in plant phenotyping, has taught me to use DNN when the problem you are trying to address is very hard, due to huge intra and/or inter class variability. This is also clear in a recent publication by Pound et al. 2017. They have a DNN performing two tasks and the one classifying the awn plants reaches 98%. But, in that case, they also ask to the network to localise the wheat spikes.

This also rises another point in this paper. I think the authors have not performed a diligent work in their literature review. The paper I mentioned is very relevant to their problem. However, there is a broad literature of DNN and plant phenotyping problems (leaf count, leaf segmentation, multi-modal images, etc.). As an example, Dobrescu et al (2017) and Giuffrida et al (2018) use ResNet, which is the same architecture the authors use in this paper. At line 320, the authors say Resnet has been used in many applications, ignoring completely all the work that has been done in plant phenotyping.

Personally, I found hard to see how DNN links with genetic analysis. I think the connection between gene and DNN output must be better explained.

At line 91, are they thinking to release the annotated images used for this paper? Because I think this is very important and if the authors do that, it would be very beneficial for the plant community.

Overall, I think the paper has merit, however it needs a lot of work to warrant a publication in Gigascience. All the arguments need to be rearranged and better explained, linking them from DNN results to genetic analysis. I also suggest to reconsider what image is supplemental and what should go in the main manuscript. Only two images are in the main manuscript, and all the rest (included tables) are as supplemental. I found this choice not very optimal.

References:

Pound et al. (2017): Deep learning for multi-task plant phenotyping
Dobrescu et al. (2017): Leveraging multiple datasets for deep leaf counting
Giuffrida et al. (2018): Phenoâ€Deep Counter: a unified and versatile deep learning architecture for leaf counting

Methods

Are the methods appropriate to the aims of the study, are they well described, and are necessary controls included? Choose an item.

Conclusions

Are the conclusions adequately supported by the data shown? Choose an item.

Reporting Standards

Does the manuscript adhere to the journal's guidelines on minimum standards of reporting? Choose an item.

Choose an item.

Statistics

Are you able to assess all statistics in the manuscript, including the appropriateness of statistical tests used? Choose an item.

Quality of Written English

Please indicate the quality of language in the manuscript: Choose an item.

Declaration of Competing Interests

Please complete a declaration of competing interests, considering the following questions:

- Have you in the past five years received reimbursements, fees, funding, or salary from an organisation that may in any way gain or lose financially from the publication of this manuscript, either now or in the future?
- Do you hold any stocks or shares in an organisation that may in any way gain or lose financially from the publication of this manuscript, either now or in the future?
- Do you hold or are you currently applying for any patents relating to the content of the manuscript?
- Have you received reimbursements, fees, funding, or salary from an organization that holds or has applied for patents relating to the content of the manuscript?
- Do you have any other financial competing interests?

- Do you have any non-financial competing interests in relation to this paper?

If you can answer no to all of the above, write 'I declare that I have no competing interests' below. If your reply is yes to any, please give details below.

I declare that I have no competing interests

I agree to the open peer review policy of the journal. I understand that my name will be included on my report to the authors and, if the manuscript is accepted for publication, my named report including any attachments I upload will be posted on the website along with the authors' responses. I agree for my report to be made available under an Open Access Creative Commons CC-BY license (http://creativecommons.org/licenses/by/4.0/). I understand that any comments which I do not wish to be included in my named report can be included as confidential comments to the editors, which will not be published.

Choose an item.

To further support our reviewers, we have joined with Publons, where you can gain additional credit to further highlight your hard work (see: https://publons.com/journal/530/gigascience). On publication of this paper, your review will be automatically added to Publons, you can then choose whether or not to claim your Publons credit. I understand this statement.

Yes Choose an item.
