# Supplementary material for: High-throughput phenotyping with deep learning gives insight into the genetic architecture of flowering time in wheat
Source: Gigascience. 2019 Nov 19;8(11):giz120. doi: 10.1093/gigascience/giz120 (PMC6862935; doi:10.1093/gigascience/giz120)
Supplement: giz120_Reviewer_1_Report_Revision_1 — Valerio Giuffrida -- 5/31/2019 Reviewed [file giz120_reviewer_1_report_revision_1.pdf]

Reviewer Report

Title: High-throughput phenotyping with deep learning gives insight into the genetic architecture of flowering time in wheat

Version: Revision 1 **Date:** 5/31/2019

Reviewer name: Valerio Giuffrida

Reviewer Comments to Author:

The paper Wang et al. presents a DNN to extract phenotype from Wheat plants. The paper has improved from its first revision and it has nice experiments merging phenotype and genotype.
I think the paper should be accepted after a few minor edits (which I highlight below). It is of great interest the authors will release the data with the paper, which enhance the value of this research for the broad community. I appreciate the efforts of the authors to release data and annotations.
Moreover, what I found interesting is the extraction of phenotype traits in the field.
My minor comments are:
Line 39-42 "Most powerfully, the deep learning approach presented here gives a conceptual advancement in high-throughput plant phenotyping as it can potentially estimate any trait in any plant species through leveraging expert knowledge from breeders, geneticist, pathologists and physiologists." I think this is a rather bold statement. Supposing that I want to predict root mass from the shoot images and suppose that I have annotated >10,000 images/plants, where I manually harvested them and took the root (dry) mass. Do the author believe it is possible to learn a DNN to create such a mapping? Of course it can be done, but I am rather skeptic about the robustness of the predictions.
Line 62-64: DL does not discovers and end-to-end process. DL optimises network parameters in an end-to-end training (most of the times). The way it is written, it seems to me that somehow DL optimises for the network architecture.
Line 66: I would say "the first filters are easily interpreted as LOW LEVEL image features"
Line 434: ResNet (or any dnn) is not restricted in the size of its input. It actually the opposite. The minimum image size resent accepts is ~300x300 (more or less). Ideally, one can provide any image size as input. The problem comes for the fully connected layers that will have millions of parameters with such a big images. Therefore, such a big network will saturate the memory of the gpu, not allowing the training. The way it is written sounds like it is a limitation of the architecture, but it is actually a (current) limitation of the hardware.
In general, I think the authors should tone down how impressive DL/CNNs. DL has been introduced in the Plant Phenotyping community since 2016. I think all the people involved in this community are aware of how DL is great in many context.
Ideas how to make this paper even better (these suggestions are not mandatory for this submission, but the authors are encouraged to follow them)
The authors make a claim from line 446 about visual scoring discrepancy. This is basically known in the literature as inter-observer (or inter-rater) variability. The authors might perform a nice study about that and check whether the machine predictions are within the human variability.

I still believe the scoring predictions should be performed as a regression task, rather than a classification task.

Methods

Are the methods appropriate to the aims of the study, are they well described, and are necessary controls included? Choose an item.

Conclusions

Are the conclusions adequately supported by the data shown? Choose an item.

Reporting Standards

Does the manuscript adhere to the journal's guidelines on minimum standards of reporting? Choose an item.

Choose an item.

Statistics

Are you able to assess all statistics in the manuscript, including the appropriateness of statistical tests used? Choose an item.

Quality of Written English

Please indicate the quality of language in the manuscript: Choose an item.

Declaration of Competing Interests

Please complete a declaration of competing interests, considering the following questions:

- Have you in the past five years received reimbursements, fees, funding, or salary from an organisation that may in any way gain or lose financially from the publication of this manuscript, either now or in the future?
- Do you hold any stocks or shares in an organisation that may in any way gain or lose financially from the publication of this manuscript, either now or in the future?
- Do you hold or are you currently applying for any patents relating to the content of the manuscript?
- Have you received reimbursements, fees, funding, or salary from an organization that holds or has applied for patents relating to the content of the manuscript?
- Do you have any other financial competing interests?
- Do you have any non-financial competing interests in relation to this paper?

If you can answer no to all of the above, write 'I declare that I have no competing interests' below. If your reply is yes to any, please give details below.

I declare that I have no competing interests

I agree to the open peer review policy of the journal. I understand that my name will be included on my report to the authors and, if the manuscript is accepted for publication, my named report including any attachments I upload will be posted on the website along with the authors' responses. I agree for my report to be made available under an Open Access Creative Commons CC-BY license (http://creativecommons.org/licenses/by/4.0/). I understand that any comments which I do not wish to be included in my named report can be included as confidential comments to the editors, which will not be published.

Choose an item.

To further support our reviewers, we have joined with Publons, where you can gain additional credit to further highlight your hard work (see: https://publons.com/journal/530/gigascience). On publication of this paper, your review will be automatically added to Publons, you can then choose whether or not to claim your Publons credit. I understand this statement.

Yes Choose an item.
