# Supplementary material for: High-throughput phenotyping with deep learning gives insight into the genetic architecture of flowering time in wheat
Source: Gigascience. 2019 Nov 19;8(11):giz120. doi: 10.1093/gigascience/giz120 (PMC6862935; doi:10.1093/gigascience/giz120)
Supplement: giz120_Reviewer_2_Report_Original_Submission — Ian Stavness, Ph.D. -- 2/24/2019 Reviewed [file giz120_reviewer_2_report_original_submission.pdf]

Reviewer Report

Title: High-throughput phenotyping with deep learning gives insight into the genetic architecture of flowering time in wheat

Version: Original Submission **Date:** 2/24/2019

Reviewer name: Ian Stavness, Ph.D.

Reviewer Comments to Author:

This study reports a high throughput phenotyping method using convolutional neural networks to automatically predict phenotypic information from field images of wheat yield plots. The first experiment used a CNN to classify wheat varieties as awn/awnless. The second experiment used a CNN to estimate breeder ratings for percentage heading. Curve-fitting to these longitudinal estimates of percentage heading was used to estimate the date of 50% heading. Predicted heading date (a proxy for flowering time in wheat) was then used to assess genetic association within the population.
This is a well executed study and well written paper. Although not the first study to apply deep learning methods to plant phenotyping, it is one of the first to demonstrate these methods in the context of plant breeding, to estimate breeder ratings with a large-scale field trial, and to associate estimated phenotypes with genomic associations. For these reasons, I believe this will be a highly influential paper in the quickly expanding research area of deep learning in plant phenotyping and breeding.
I have a few suggestions to help contextualize the work and clarify some aspects of the methodology. I consider these minor revisions.
1) Better contextualize the study with respect to previous work in deep learning for plant phenotyping:
As currently written, the introduction suggests this is the first paper in the area of deep learning in plant phenotyping: "we hypothesized that this deep learning approach could be an equally powerful tool for scoring subtle, complex phenotypic differences directly from images in segregating plant populations". I suggest including additional previous work in the introduction/discussion:
- Move citation [1] from the discussion up to the introduction;
- The introductory remark that "'complex traits … cannot be assessed by a linear function of pixel value" has already been discussed in [2] (in particular, see figure 1) and warrants a citation; and
- Other deep learning studies in wheat should also be mentioned, either in introduction or discussion, e.g. [3-5]
This will help the authors to differentiate the novelty of their work, which is substantial (outdoor images, large scale trials, directly related to plant breeder ratings, etc.), instead of simply referring to general image classification papers.
2) Awn classification task:
As a computer vision task, the binary awn/awnless classification problem seems fairly straight forward, and therefore the high accuracy is not surprising. The point about 'MFA-2018' having a heterogeneous and 'atypical awnlette' pattern is interesting, and perhaps could be illustrated by including an example image from this variety to Figure S2.
(Note, that it is common to report precision/recall for these types of tasks. This is probably fine to omit

due to the high accuracy; however these additional metrics can be useful for other researchers to reproduce/extend the work.)

3) Measurement of percentage heading section:

The description of this task could be revised for clarity. The difference between "percentage heading" score and "heading date" score should be clarified. Are the breeder scores done by visual inspection of the plots in the field or by inspection of images (I think from the field, but it would be helpful to be explicit here)? How frequent are "percentage heading" scores and images captured (this is clear in Fig. 2, but could be stated in the text)? I am also curious about how "heading date" is scored â€” are plots visited every day in order to find the precise 50% heading date?

From Fig. 2, It looks like the visual scores for "percentage heading" and the images were not captured on the same dates â€” how is this discrepancy handled when generating training/testing datasets?

I think it might help to use consistent and different terms for what's done by the breeder vs. computer, e.g. use "score/scoring" as the task being done by the breeder, and "estimation/classification" as the task being done by the CNN.

Line 134: "Due to tightly closed flowers in wheat, spike emergence (heading time or heading date) is used as a close proxy for breeding and genetics." â€” "proxy for breeding" is confusing, I suggest revising to "spike emergence is used as a close proxy for flowering time in breeding and genetics studies"

Line 135: "Observing initial proof of concept for using deep learning to score a simple Mendelian morphological trait" â€” this sentence is a bit awkward. I think you are saying that the first study on awn/awnless classification was a proof of concept that gave you confidence to extend the CNN approach to a more complex trait of percentage heading, but this is not clear. Perhaps this should be the opening sentence of the section to bridge from the previous section?

4) Clarify the loss function used for heading classification:

The authors develop a custom loss function that is tailored to the misclassification rates in the dataset for percentage heading. In the methods section, they state "In our data, the percentage of the mislabeled images which has offset 10% is about 10% in each class, and the mislabeled images which has offset 20% is about 5% in each class." How were these misclassification rates discovered? Was an intra/inter-rater reliability study conducted? Are these actual misclassifications, or is it moreso that some of the classes are ambiguous or difficult to reliably distinguish? Is it all classes, or are the misclassifications predominantly in low heading or high heading classes? It is also not clear how important this custom loss function is â€” a comparison to a standard cross-entropy loss would be helpful here. Is this custom loss function generally applicable other human-rater labels? How were the hyper parameters ("0.7 for correct class, 0.1 for 10% off and 0.05 for 20% off") for the loss function determined?

5) Other suggestions:

Line 323: training procedure â€” it would be helpful to state how the validation set was used in training, e.g. was it used to assess hyper-parameters, or number of training epochs, etc.? A supplemental figure showing training/validation loss vs. time is usually illustrative.

Figure S3: double-check the sub-figure placement, I believe that "test" should be the bottom sub-figure corresponding to (c), but it looks to be the top image corresponding to (a) presently.

Code availability: in order for other researchers to reproduce the work, it would be helpful if the code were made available along with the dataset.

References

[1] Pound, M. P., Atkinson, J. A., Townsend, A. J., Wilson, M. H., Griffiths, M., Jackson, A. S., ... & Pridmore, T. P. (2017). Deep machine learning provides state-of-the-art performance in image-based plant phenotyping. Gigascience, 6(10), gix083.
[2] Ubbens, J. R., & Stavness, I. (2017). Deep plant phenomics: a deep learning platform for complex plant phenotyping tasks. Frontiers in plant science, 8, 1190.
[3] Aich, S., Josuttes, A., Ovsyannikov, I., Strueby, K., Ahmed, I., Duddu, H. S., ... & Stavness, I. (2018, March). Deepwheat: Estimating phenotypic traits from crop images with deep learning. In 2018 IEEE Winter Conference on Applications of Computer Vision (WACV) (pp. 323-332). IEEE.
[4] Hasan, M. M., Chopin, J. P., Laga, H., & Miklavcic, S. J. (2018). Detection and analysis of wheat spikes using Convolutional Neural Networks. Plant methods, 14(1), 100.
[5] Pound, M. P., Atkinson, J. A., Wells, D. M., Pridmore, T. P., & French, A. P. (2017). Deep learning for multi-task plant phenotyping. In Proceedings of the IEEE International Conference on Computer Vision (pp. 2055-2063).

Methods

Are the methods appropriate to the aims of the study, are they well described, and are necessary controls included? Choose an item.

Conclusions

Are the conclusions adequately supported by the data shown? Choose an item.

Reporting Standards

Does the manuscript adhere to the journal’s guidelines on minimum standards of reporting? Choose an item.

Choose an item.

Statistics

Are you able to assess all statistics in the manuscript, including the appropriateness of statistical tests used? Choose an item.

Quality of Written English

Please indicate the quality of language in the manuscript: Choose an item.

Declaration of Competing Interests

Please complete a declaration of competing interests, considering the following questions:

- Have you in the past five years received reimbursements, fees, funding, or salary from an organisation that may in any way gain or lose financially from the publication of this manuscript, either now or in the future?
- Do you hold any stocks or shares in an organisation that may in any way gain or lose financially from the publication of this manuscript, either now or in the future?
- Do you hold or are you currently applying for any patents relating to the content of the manuscript?
- Have you received reimbursements, fees, funding, or salary from an organization that holds or has applied for patents relating to the content of the manuscript?
- Do you have any other financial competing interests?
- Do you have any non-financial competing interests in relation to this paper?

If you can answer no to all of the above, write 'I declare that I have no competing interests' below. If your reply is yes to any, please give details below.

I declare that I have no competing interest

I agree to the open peer review policy of the journal. I understand that my name will be included on my report to the authors and, if the manuscript is accepted for publication, my named report including any attachments I upload will be posted on the website along with the authors' responses. I agree for my report to be made available under an Open Access Creative Commons CC-BY license (http://creativecommons.org/licenses/by/4.0/). I understand that any comments which I do not wish to be included in my named report can be included as confidential comments to the editors, which will not be published.

Choose an item.

To further support our reviewers, we have joined with Publons, where you can gain additional credit to further highlight your hard work (see: https://publons.com/journal/530/gigascience). On publication of this paper, your review will be automatically added to Publons, you can then choose whether or not to claim your Publons credit. I understand this statement.

Yes Choose an item.
