# Supplementary material for: High-throughput phenotyping with deep learning gives insight into the genetic architecture of flowering time in wheat
Source: Gigascience. 2019 Nov 19;8(11):giz120. doi: 10.1093/gigascience/giz120 (PMC6862935; doi:10.1093/gigascience/giz120)
Supplement: giz120_Supplemental_Files [file REVIEW-RESPONSE-2_GigaScience_20190628.pdf › REVIEW-RESPONSE-2_GigaScience_20190628.pdf]

REVIEW RESPONSE

Reviewer reports:
Reviewer #1: The paper Wang et al. presents a DNN to extract phenotype from Wheat plants. The paper has improved from its first revision and it has nice experiments merging phenotype and genotype.
I think the paper should be accepted after a few minor edits (which I highlight below). It is of great interest the authors will release the data with the paper, which enhance the value of this research for the broad community. I appreciate the efforts of the authors to release data and annotations. Moreover, what I found interesting is the extraction of phenotype traits in the field.

My minor comments are:

Line 39-42 "Most powerfully, the deep learning approach presented here gives a conceptual advancement in high-throughput plant phenotyping as it can potentially estimate any trait in any plant species through leveraging expert knowledge from breeders, geneticist, pathologists and physiologists." I think this is a rather bold statement. Supposing that I want to predict root mass from the shoot images and suppose that I have annotated >10,000 images/plants, where I manually harvested them and took the root (dry) mass. Do the author believe it is possible to learn a DNN to create such a mapping? Of course it can be done, but I am rather skeptic about the robustness of the predictions.

This is a very good point and clearly a vague and overstated sentence on our part. We do not think that it would be possible to predict root mass from shoot images or something like this. Currently, this would be much more limited to traits for which the combination of both high resolution images and breeder scores are obtained for the same trait. We have edited the abstract with less bold language to hopefully clarify this point and the limitations.

"The deep learning approach presented here gives a conceptual advancement in high-throughput plant phenotyping as it can potentially estimate any trait in any plant species for which the combination of breeder scores and high-resolution images can be obtained, capturing the expert knowledge from breeders, geneticist, pathologists and physiologists to train the networks.

Line 62-64: DL does not discovers and end-to-end process. DL optimizes network parameters in an end-to-end training (most of the times). The way it is written, it seems to me that somehow DL optimizes for the network architecture.

This is a good assessment of the vernacular and we have change accordingly to 'optimizes'.
"Instead deep learning optimizes a complete end-to-end process to map data samples to outputs…"

Line 66: I would say "the first filters are easily interpreted as LOW LEVEL image features"

We have added 'low level'.

Line 434: ResNet (or any dnn) is not restricted in the size of its input. It actually the opposite. The minimum image size resent accepts is ~300x300 (more or less). Ideally, one can provide any image size as input. The problem comes for the fully connected layers that will have millions of parameters with such a big images. Therefore, such a big network will saturate the memory of the gpu, not allowing the training. The way it is written sounds like it is a limitation of the architecture, but it is actually a (current) limitation of the hardware.

We have clarified this sentence to state that it is indeed the hardware and not the network architecture that limits the image size being used.
Updated: "Resnet, and most convolutional neural networks, are presently restricted in the size of the image they can analyze due to current hardware limitations (e.g. GPU memory) used for training the networks."

In general, I think the authors should tone down how impressive DL/CNNs. DL has been introduced in the Plant Phenotyping community since 2016. I think all the people involved in this community are aware of how DL is great in many context.

We have worked through the abstract, introduction and conclusion sections to moderate the language used to describe the CNNs and the results.

Ideas how to make this paper even better (these suggestions are not mandatory for this submission, but the authors are encouraged to follow them)
The authors make a claim from line 446 about visual scoring discrepancy. This is basically known in the literature as inter-observer (or inter-rater) variability. The authors might perform a nice study about that and check whether the machine predictions are within the human variability.

We agree this would be a nice complimentary study. Unfortunately during the collection this this data we did not have the personnel time to collect duplicate datasets to assess the inter- and intra-rater variability.

I still believe the scoring predictions should be performed as a regression task, rather than a classification task.

While the Deep Learning paradigm can be trained for regression, the consensus in the field is that single variable regression problems are often done better by discretizing (binning) the output variable and doing classification into the discrete classes. This minimizes the effect of noisy or outlier datapoints, makes training the deep network more efficient, and across many problem domains gives substantially better results.

Work that follows this approach include age-estimation:
Niu, Zhenxing, et al. "*Ordinal regression with multiple output CNN for age estimation.*" Proceedings of the IEEE Conference on Computer Vision and Pattern Recognition. 2016.

and depth estimation from imagery:
Cao, Yuanzhouhan, Zifeng Wu, and Chunhua Shen. "*Estimating depth from monocular images as classification using deep fully convolutional residual networks*." IEEE Transactions on Circuits and Systems for Video Technology28.11 (2017): 3174-3182.

One important consideration in training the network for percentage heading as a classification task rather than regression is that the percentage heading is in reality an ordinal scale with bounds. Therefore, is it is not truly a regression task. For example, training a network that could output values such as -10% or 105% heading does not accord with the biological reality. Furthermore, the bins at 10% intervals accords with the visual scoring that we done in 11 discrete bins (0%, 10%, 20% …. 100%) rather than on a continuous scale.
